# Building geodemographic regions: commuting, productivity and uneven spatial development in England and Wales

**DOI:** 10.1080/00343404.2025.2485132

**Published:** 2025-05-06

**Authors:** Stephen Hincks, Hadi Arbabi, Ruth Hamilton

**Affiliations:** a School of Geography and Planning, University of Sheffield, Sheffield, UK; b School of Mechanical, Aerospace and Civil Engineering, University of Sheffield, Sheffield, UK

**Keywords:** geodemographic, commuting, functional regions, settlement scaling, productivity, regional inequalities, J1, J6, J61, R1, R2, R23

## Abstract

We develop and apply a novel geodemographic classification of commuting flows to delineate 486 functional labour market areas (LMAs) across six commuter groups in England and Wales. Framed by the north–south divide, we then use settlement scaling to examine how economic and infrastructural agglomeration influence productivity, using the geodemographic LMAs as our base units. We find that disparities in mobility and infrastructure contribute to spatial productivity differences, with poorer intra-city connectivity in northern regions. Even among LMAs with similar commuter profiles, productivity diverges across the divide, highlighting how economic and infrastructural inequalities reinforce commuting interactions and regional productivity gaps.

## 1. INTRODUCTION

Functional approaches to regionalisation have a long and varied history in regional studies, where administrative boundaries are considered to lack sensitivity to functional interactions (Brown & Hincks, 2008; Casado-Díaz, 2000; Smart, 1974). For Brown and Holmes (1971, p. 57), functional regions consist of areas or locational entities with stronger internal interactions than with outside areas. This feature is attractive to economic policymakers because it allows for internalising policy interventions in areas reflecting *daily* home–work interactions and limited spatial spillovers (Brown & Hincks, 2008; Casado-Díaz et al., 2017; Hincks, 2012; Martínez-Bernabeu et al., 2020).

This paper focuses on delineating commuting-based functional regions as approximations to labour market areas (LMAs). We contribute to debates on LMA delineation and application in economic planning and regional studies by addressing a longstanding question: How do the commuting behaviours of different workforce groups affect the geography, structure and productivity of functional LMAs? (Van der Laan & Schalke, 2001; Karlsson & Olsson, 2006; Office for National Statistics (ONS), 2016). This question is significant because different groups of workers adopt varied commuting behaviours to overcome spatial and structural mismatches between home and workplace locations, impacting mobility and productivity (Arbabi et al., 2019; Hincks, 2012; Shen & Batty, 2019).

In several studies, this has prompted the delineation of subgroup LMAs using commuting flows that are disaggregated by individual commuter characteristics such as age, sex or socio-economic status (Casado-Díaz, 2000; Farmer & Fotheringham, 2011; Green et al., 1986; ONS, 2016; Shen & Batty, 2019). This process of isolating individual traits from aggregate flows allows variations in the commuting behaviours of workers with a shared characteristic to be reflected in LMA structures.

As an extension to these studies, the contribution of this paper lies in its definition of *geodemographic*-based subgroup LMAs for the first time, using commuting-flow data for England and Wales. Geodemographic systems classify georeferenced data into homogeneous groups based on various demographic and socio-economic attributes (Vickers & Rees, 2011). Employing a method from Hincks et al. (2018), we establish a new geodemographic flow-based classification for England and Wales, segmenting commuters by 49 demographic and socio-economic traits. Using the Intramax hierarchical grouping algorithm, commonly used in functional regionalisation exercises (Brown & Hincks, 2008; Masser & Brown, 1975), we define geodemographic-based LMAs for England and Wales, where different combinations of demographic and socio-economic characteristics, and modal choices are reflected in the structures of subgroup LMAs. Through the lens of settlement scaling, we then offer a novel analysis of economic and infrastructure agglomeration effects using these new LMAs as our spatial unit, contributing to debates on productivity and regional spatial inequalities, exemplified in England and Wales (Martin et al., 2016; McCann, 2016; O’Brien et al., 2019).

## 2. LMAs, COMMUTING AND GEODEMOGRAPHICS

The starting point for our conceptualisation is the idea that functional LMAs represent spatial units that reflect the relationship between labour supply and demand. In labour markets, workers sell their effort while retaining their inherent capital, expressing labour production and consumption through job, employer, and home–work preferences (Green, 1997). As a result, labour markets develop distinctive characteristics, structures and dynamics that result from the institutional reproduction and social regulation of labour.

Commuting is one expression of the production and consumption of labour that results in a demand–supply relationship that is expressed over geographical space (Hincks, 2012). In LMA delineation, consideration of *daily* travel-to-work patterns feature prominently (Casado-Díaz, 2000). Two frameworks underpin functional LMA delineation. The first stresses the homogeneity of LMAs as ‘spatially limited entities, within which aggregated supply and demand meet’ (Van der Laan & Schalke, 2001, p. 203). This framework assumes that workers will seek to minimise commuting to balance home and workplace locations, resulting in bounded labour markets in which most residents will seek both housing and employment opportunities (Smart, 1974).

The second framework is rooted in a heterogeneous view of the labour market that ‘stresses the existence of submarkets of types of labour’ (Van der Laan & Schalke, 2001, p. 203). Rather than focusing on the minimisation of commuting costs, this framework acknowledges that labour is segmented, leading to tensions in balancing residential and workplace locations that potentially increase journey times and/or distances (Green, 1997). While workers will often seek to minimise commuting costs, it is also recognised that they will accept a wide range of combinations of residential and workplace locations in trading-off commuting costs (Acheampong, 2020; Green, 1997; Hincks & Wong, 2010).

The discontinuities in the balance between residential and workplace locations have been shown to extend to a range of demographic and socio-economic characteristics. As education, income and socio-economic status increase, so workers tend to commute longer distances and/or times (Green, 1997). Women have tended to adopt shorter commutes than men (McQuaid & Chen, 2012); and younger and older groups also tend to have shorter commutes. The effect of ethnicity on commuting has long been a point of contention in the UK. Thomas (1998) found that ethnic minority groups have shorter commutes than white workers, while McQuaid and Chen (2012) found that ethnicity affected the time spent commuting, but only for men employed full-time. Lucas et al. (2016) found that while commuting distances were similar for white and non-white commuters in the UK – treated in their models as a binary variable – non-white commuters make fewer trips per week, which they suggest might be due to greater public transport use and more local travel patterns. In addition, car-based mobility has contributed to extended commuting patterns, but it has also been suggested that longer distance commuting is conditioned by access to public transport networks (McQuaid & Chen, 2012). What emerges here is an understanding of commuting as a suboptimal process, leading to segmented LMAs driven by labour supply and demand (Coombes et al., 1988).

In the UK, travel-to-work areas (TTWAs), defined using census-derived commuting flows, serve as proxy LMAs with labour supply-and-demand expressed through commuting patterns (Coombes & Openshaw, 1982; Hincks & Wong, 2010; Smart, 1974). The UK’s TTWA framework sets a minimum self-containment at 75% for TTWAs with a minimum residential workforce of 3500, while those TTWAs with over 20,000 residents require at least 70% self-containment (Coombes, 1998). Contiguity constraints are imposed on TTWA delineation so that base units near one another are grouped to form ‘coherent’ geographies (Coombes et al., 1986).

Although TTWAs are typically developed using aggregated commuting flows, labour market segmentation has also been reflected in the delineation of TTWAs since 1981, when aggregated Census of Population commuting matrices were subsetted along male, female and socio-economic lines (Coombes et al., 1988; Green et al., 1986). More recently, the ONS released a suite of ‘Alternative Travel-to-Work Areas’ derived from 2011 Censuses of the UK nations,^1^ exposing differences in TTWA structures and geographies across the UK, based on individual demographic, socio-economic or modal traits. Similarly, Casado-Díaz (2000) in Spain and Farmer and Fotheringham (2011) in Ireland have identified subgroup LMAs, highlighting differences in geography, size and the number of LMAs, which were influenced by variations in individual demographic, socio-economic and modal characteristics.

Underlying this work is the impetus to understand ‘how the structure of aggregate functional regions reflects the intricacies of subgroup commuting behaviour’ (Farmer & Fotheringham, 2011, p. 2739). Where recent work focuses on single demographic and socio-economic attributes, we offer a novel alternative that involves defining LMAs using geodemographic-based flow-data in which commuters are partitioned based on shared characteristics across multiple attributes.

## 3. METHODOLOGY AND DATA

Considering the context above, this section details the approach adopted to delineate subgroups LMAs for England and Wales.

### 3.1. Step 1: Developing a geodemographic classification of commuting flows

First, we developed a new classification of commuting flows for England and Wales using origin–destination special workplace statistics (SWS) from the 2011 Census, available at the level of middle layer super output areas (MSOAs).^2^ We employ a raw commuting dataset originally compiled by Hincks et al. (2018) in their classification of commuting flows for England and Wales.

Rather than adopting their original classification, we chose to rerun the process to undertake additional sensitivity testing before developing our new set of geodemographic LMAs. Our sensitivity testing involved two main considerations. Hincks et al. (2018) note that a limitation of *k*-means clustering is that case order can affect the outcome of the cluster solution. We focused here on extending the number of iterations of the cluster runs to minimise case order effects (see below). In addition, we adopted the *k*-means++ algorithm with the aim of improving the cluster initialisation, due its efficiency in converging to a local optimum and owing to its enhanced performance over conventional *k*-means (Arthur & Vassilvitskii, 2007).

In deriving our commuting classification, we closely follow the methodology employed by Hincks et al. (2018), beyond the variations noted above. In the raw commuting dataset, the total number of commuters within each MSOA interaction (e.g., E02000001 → E02000119) formed the numerator and each characteristic variable (e.g., male, age 16–24) formed denominators.

Flows of five people or fewer on the numerator variable were removed because this led to improved distributions of many of the variables following transformation and standardisation and served to minimise the effects of small cell disclosure control used to protect the anonymity of individuals (Hincks et al., 2018; Stillwell & Duke-Williams, 2007). While the removal of small flows improved the distribution of variables, it resulted in uneven effects across commuter categories, influencing the underlying classification. For example, 63% of car commuting flows had magnitudes of commuters of between one and five individuals, compared with 5% for motorcycles and 7% for walking. Longer distance flows often had fewer individuals, while shorter distance commutes exceeded the five-person threshold more frequently.

The final dataset captures 513,892 commuting interactions, representing 18.4 million of the 26.5 million workers (70%) recorded in the 2011 Censuses of England and Wales. It includes 49 demographic, socio-economic and modal variables (Table 1), reduced from an initial 89. Following Hincks et al. (2018), we employed visual analysis of outliers, normality testing and Pearson correlation to evaluate candidate variables to minimise data redundancy. For the Pearson correlation, a threshold of ±0.70 was adopted (Hincks et al., 2018), which lies between the ±0.90 suggested by Mooi and Sarstedt (2011) and the ±0.60 used by Gale et al. (2016) in their development of a geodemographic classification of UK census geographies.

**Table 1.** Commuter categories and variables.

Category	Variable	Radial chartreference^b^	Mean(%)^c^	SD
Sex	Male/female^a^	1	51.6%	20.1
Ethnic group	White/non-White^a^	2		
Age (years)	16–24	3	12.3%	11.5
	25–34	4	24.1%	16.3
	35–49	5	37.1%	16.0
	50–64	6	23.9%	14.6
Method of travel to work	Train	7	7.0%	18.0
	Bus, minibus or coach	8	8.4%	13.1
	Driving/passenger in a car or van	9	65.9%	29.0
	Bicycle	10	2.3%	5.3
	On foot	11	5.1%	11.1
National Statistics Socio-economic Classification (NS-Sec)	Higher managerial and administrative occupations	12	3.5%	6.2
	Higher professional occupations	13	11.3%	13.4
	Lower professional and higher technical occupations	14	18.7%	14.9
	Lower managerial and administrative occupations	15	6.7%	8.2
	Higher supervisory occupations	16	3.6%	6.0
	Intermediate occupations	17	15.7%	12.3
	Lower supervisory occupations	18	4.4%	6.4
	Lower technical occupations	19	3.8%	6.6
	Semi-routine occupations	20	13.8%	12.8
	Routine occupations	21	10.3%	11.8
Industry	Manufacturing	22	9.8%	14.6
	Construction	23	5.0%	8.3
	Wholesale, retail trade; repair of motor vehicles	24	15.7%	15.3
	Transport and storage	25	5.2%	9.9
	Accommodation and food service activities	26	4.9%	8.4
	Financial and insurance activities	27	4.3%	10.0
	Professional, scientific and technical activities	28	6.3%	10.1
	Administrative and support service activities	29	4.2%	7.1
	Public administration, defence; social security	30	6.6%	7.1
	Education	31	11.8%	15.3
	Human health and social work activities	32	14.3%	17.8
Occupation	Managers, directors and senior officials	33	11.8%	11.1
	Professional occupations	34	20.8%	17.9
	Associate professional and technical occupations	35	13.0%	12.6
	Administrative and secretarial occupations	36	12.3%	11.1
	Skilled trades occupations	37	8.5%	10.2
	Caring, leisure and other service occupations	38	9.6%	11.6
	Sales and customer service occupations	39	7.7%	10.4
	Process, plant and machine operatives	40	7.3%	10.7
	Elementary occupations	41	9.7%	11.4
Hours worked	Part-time: ≤ 15 h	42	7.3%	9.1
	Part-time: 16–30 h	43	17.7%	13.8
	Full-time: 31–48 h	44	62.8%	17.9
	Full-time: ≥ 49 h	45	12.2%	11.9
Approximated social grade	Approximated social grade AB	46	27.7%	20.0
	Approximated social grade C1	47	32.3%	16.1
	Approximated social grade C2	48	21.3%	14.8
	Approximated social grade DE	49	18.5%	15.8

Note: ^a^Calculated for the retained ‘male’ variable. The remaining 48.4 reflects the excluded ‘female’ variable.

^b^See Figure A2 in Appendix A in the supplemental data online.

^c^Means might not sum to 100% for each category owing to the exclusion of skewed/correlated variables.

The 49 variables were then subjected in *R* to *k*-means clustering, an unsupervised machine learning technique that aims to minimise within-group variations and maximise variations between groups. Following Hincks et al. (2018), flows are iteratively reassigned to clusters to identify a suite of centroids that minimise:

(1) $$V=\overset{y=1}{\underset{k}{\sum }}\overset{x=1}{\underset{v}{\sum }}\overset{i=1}{\underset{n_{k}}{\sum }}(z_{\mathrm{yxi}}-\;\mu_{\mathrm{xy}})\;$$

where *V* is the sum of squared distances of all variables from cluster means for all clusters, *z*_*xyi*_ is the standardised variable for flow *i*, variable *x* and cluster *y*, *μ*_*yx*_ is the mean for variable *x* in cluster *y*, *k* is the number of clusters, *v* is the number of variables, and *n*_*k*_ is the number of flows in the cluster.

In *k*-means clustering, there are no fixed criteria for determining optimal cluster solutions, although various diagnostic procedures have been proposed (Charrad et al., 2014). Moreover, the case order of observations in the original dataset can affect the outcome of cluster solutions. To mitigate case order effects, cluster solutions were iterated using randomly ordered cases (flows) (Gale et al., 2016). This study focuses on deriving a single-tier commuting classification with *n* clusters, constrained within a range of three to nine groups (Hincks et al., 2018), with cluster initialisation undertaken through the *k*-means++ algorithm.

Cluster solutions were rerun 1000 times for each group configuration (*n* = 3–9), resulting in a final set of 7000 cluster solutions. In this study, the optimal solution was determined as the run that minimises the within-cluster sum of squares (WCSS) statistic. WCSS measures the proximity of objects within each cluster solution to the centroid, indicating cluster homogeneity (Gale et al., 2016, p. 10). Tukey post-hoc tests assessed cluster distances to determine if the distances between cluster centroids were statistically significant and warranted their separation as distinct groups.

### 3.2. Step 2: Delineating geodemographic LMAs

#### 3.2.1. The Intramax regionalisation approach

Having clustered the flows, we subject the segmented flows to a regionalisation procedure to delineate LMAs for each group. There is no ‘natural’ method for delineating LMAs (Coombes, 1995, pp. 46–47) but three classes of procedure are commonly used: hierarchical clustering (e.g., Masser & Brown, 1975), multistage aggregation (Coombes et al., 1986) and central place aggregation (Karlsson & Olsson, 2006). A fourth-class, using network and community detection, has emerged recently (Farmer & Fotheringham, 2011; Hamilton & Rae, 2020).

We employ the Intramax procedure, a hierarchical regionalisation algorithm developed by Masser and Brown (1975), to summarise flow structures in interaction matrices (Brown & Hincks, 2008). Masser and Scheurwater (1980) note the merits of Intramax over more computationally complex functional distance and iterative proportional fitting procedures (Brown & Hincks, 2008, p. 2231). Here Intramax aims to maximise the proportion of total interaction occurring within aggregations of base units in the cross-diagonal of an interaction matrix, while minimising system-wide cross-boundary flows (Masser & Brown, 1975, p. 510). A modified version of Ward’s (1963) hierarchical aggregation procedure, Intramax focuses on relative interaction strength after accounting for size variation in row and column totals (Brown & Hincks, 2008, p. 2231). These aspects are reflected in the objective function specification, maximised at each aggregation step to represent the difference between observed and expected flows, calculated by standardising the matrix to sum to unity (Brown & Hincks, 2008). Exceeding an expected value indicates a level of interaction higher than anticipated.

Interactions between pairs of base units are then evaluated and where the difference between observed and expected interactions are greatest, then the pair of base units are combined. After the fusion, row and column totals are re-estimated before the search begins for the next pair of areas for which the objective function is maximised (Brown & Hincks, 2008, p. 2231). Following Brown and Pitfield (1990, p. 62) and Brown and Hincks (2008, p. 2231) the objective function is expressed as:

(2) $$\mathrm{MaxZ}=\frac{a(i,\;j)}{a{\left(i,\;j\right)}^{*}}+\frac{a(\;j,\;i)}{a{\left(\;j,\;i\right)}^{*}},\;i\neq j\;\;\;\;\;\;\;\;\;$$

where *a*(*i,j*) is the observed value of the flow in the *i*th row and the *j*th column of the interaction matrix following standardisation, where:

(3) $$\underset{i}{\sum }\underset{j}{\sum }a(i,\;j)=1\;\;\;\;\;\;\;\;\;\;\;\;\;$$

and where expected values are ( )* are estimated as:

(4) $$a(i,\;j{)}^{*}=\underset{p}{\sum }a(\;p,\;j)\underset{q}{\sum }a(i,\;q)\;\;\;\;\;\;\;\;\;$$

(5) $$a(\;j,\;i{)}^{*}=\underset{p}{\sum }a(\;p,\;i)\underset{q}{\sum }a(\;j,\;q)$$

subject to a contiguity constraint:

ci,j=1whenbaseunitsiandjarecontiguous

(6) ci,j=0whenbaseunitsiandjarenon-contiguous

Aggregation proceeds through a stepwise process, where each MSOA starts out as a single base unit and through aggregation is combined until all MSOAs are fused so that only one group exists – in our case covering the whole of England and Wales. Here the aggregation of non-adjacent base units is avoided by adopting the contiguity constraint above (6).

#### 3.2.2. Evaluating regionalisation solutions

The next step involved identifying criteria to evaluate LMA solutions that are produced at each stage of the stepwise procedure. Here, we draw on and adapt recent approaches to define such criteria (Casado-Díaz et al., 2017; Martínez-Bernabeu et al., 2020), underpinned by four principles (Martínez-Bernabeu et al., 2020, p. 742).

The first is *autonomy*, which seeks to maximise the internalisation of commuting flows for each individual LMA. We measure autonomy using median self-containment measures for both supply- and demand-side criteria, targeting a median self-containment of 70% against which the solutions at each step of the aggregation process are assessed. This is calculated as:

(7) $$\mathrm{mdn}\left(\frac{\mathrm{Fa},\;a}{\mathrm{Ra}},\;\frac{\mathrm{Fa},\;a}{\mathrm{Wa}},\;0.70\right)\;\;\;\;\;\;\;\;\;\;$$

where *Fa*,*a* is the number of people who both live and work in the area concerned; *Ra* is the number of workers living in the area concerned (demand-side); and *Wa* is the number of people who work in the area concerned (supply-side) (Coombes, 1998). Additionally, all LMAs must meet a minimum self-containment threshold of 60%, expressed as:

(8) $$\min\left(\frac{\mathrm{Fa},\;a}{\mathrm{Ra}},\;\frac{\mathrm{Fa},\;a}{\mathrm{Wa}},\;0.60\right)\;\;\;\;\;\;\;$$

The aim is to ensure that each LMA not only contributes to a high median self-containment but also maintains a baseline level of autonomy.

The second criterion is *homogeneity*, which aims to minimise the range of LMA sizes. Following Martínez-Bernabeu et al. (2020), our measure of homogeneity is the working population size of each LMA, which we seek to minimise. The third criterion is *balance*, which aims to balance labour supply and demand in each LMA, where more balanced LMAs are considered to have shorter average commuting distances. Here Martínez-Bernabeu et al. adopt a measure of jobs balance (*B*) for each LMA, defined as the ‘ratio between the number of jobs at local workplaces [*Wa*] and its number of employed residents [*Ra*]’ (p. 744), expressed as:

(9) $$B_{\mathrm{LMA}}=\frac{\mathrm{Wa}}{\mathrm{Ra}}\;\;\;\;\;\;\;\;\;\;\;\;\;\;\;\;$$

The final criterion identified by Martínez-Bernabeu et al. is *cohesion*. Here we include the number of LMAs in recognition that larger functional regions may exhibit lower cohesion. In our study, we considered the number of LMAs at each stage of the aggregation procedure to identify notable ‘steps’ in the aggregation profile within each grouping solution produced by the Intramax run (Martínez-Bernabeu et al., 2020). We do this in our approach by prioritising the maximisation of the number of LMAs.

In the final step, we use these criteria to identify the ‘optimal’ solutions for each geodemographic group, using the concept of maximum entropy to guide the process. Entropy here represents the uncertainty or randomness in the distribution of the four key criteria: median supply-side self-containment, median demand-side self-containment, homogeneity, and balance. A bespoke Python script is used to filter the LMA solutions at different aggregation steps, ensuring that both supply- and demand-side self-containment meet or exceed a 60% threshold, while also prioritising the maximisation of LMAs through the cohesion criterion.

Once the solutions are filtered, we compute the maximum entropy (Shannon, 1948) for each criterion at each step of the Intramax aggregation process. Entropy is calculated using Python’s scipy.stats.entropy function, expressed as:

(10) $$H(P)=\;-\;\overset{n}{\underset{i=1}{\sum }}p_{i}\log\;P_{i}\;\;\;\;\;\;\;\;\;\;\;\;\;$$

where $p_{i}$ represents the normalised values of the four metrics across the criteria for each LMA solution. The normalisation function converts the raw scores into a probability distribution. Each metric is weighted equally – to reflect the relative balance among the criteria – and summed to calculate a measure of total maximum entropy.

The measure of maximum entropy is then used to identify break points, indicating significant changes in the distribution. Higher entropy suggests greater variability, while lower entropy signals more uniformity. In this context, identifying the point where total entropy is maximised helps capture natural divisions in the data. To find break points, the distribution is divided into five bins. We evaluate potential break points by splitting the data into two segments at each point and calculating the entropy of both. The total entropy for any given break point is the sum of the entropies of the two segments. By comparing total entropy across all break points, we then identify the point where the most significant change in distribution occurs.

## 4. GEODEMOGRAPHIC CLASSIFICATION OF COMMUTING FLOWS FOR ENGLAND AND WALES

Through a combination of WCSS and analyses of variance (ANOVAs), a six-cluster solution was identified as the optimal group configuration for the commuting flows.^3^ It is not possible here to summarise the diagnostic results for all cluster configurations given that 7000 cluster runs were undertaken. Instead, the best performing configurations for each solution (*n* = 3–9) are summarised in Table 2.

**Table 2.** Summary of diagnostic statistics to determine the optimum cluster solution (*n* = 3–9).

Solution	Levene statistic	Within-cluster sum of squares (WCSS) statistic	Tukey statistic^a^
3	9040.610, d.f. 2, d.f. 513,889, *p* < 0.000	38,635.769, d.f. 513,889, *p* < 0.000	Yes
4	5383.399, d.f. 3, d.f. 513,888, *p* < 0.000	38,986.743, d.f. 513,888, *p* < 0.000	Yes
5	4428.927, d.f. 4, d.f. 513,887, *p* < 0.000	38,453.633, d.f. 513,887, *p* < 0.000	Yes
6	2595.849, d.f. 5, d.f. 513,886, *p* < 0.000	36,768.227, d.f. 513,886, *p* < 0.000	Yes
7	2506.438, d.f. 6, d.f. 513,885, *p* < 0.000	36,877.761, d.f. 513,885, *p* < 0.000	No
8	2481.374, d.f. 7, d.f. 513,884, *p* < 0.000	36,885.448, d.f. 513,884, *p* < 0.000	No
9	1561.793, d.f. 8, d.f. 513,883, *p* < 0.000	36,791.546, d.f. 513,883, *p* < 0.000	Yes

Note: ^a^Calculates whether distances of cases from the classification cluster centre are significant based on the mean difference at the 0.05 level.

**Figure 1.**
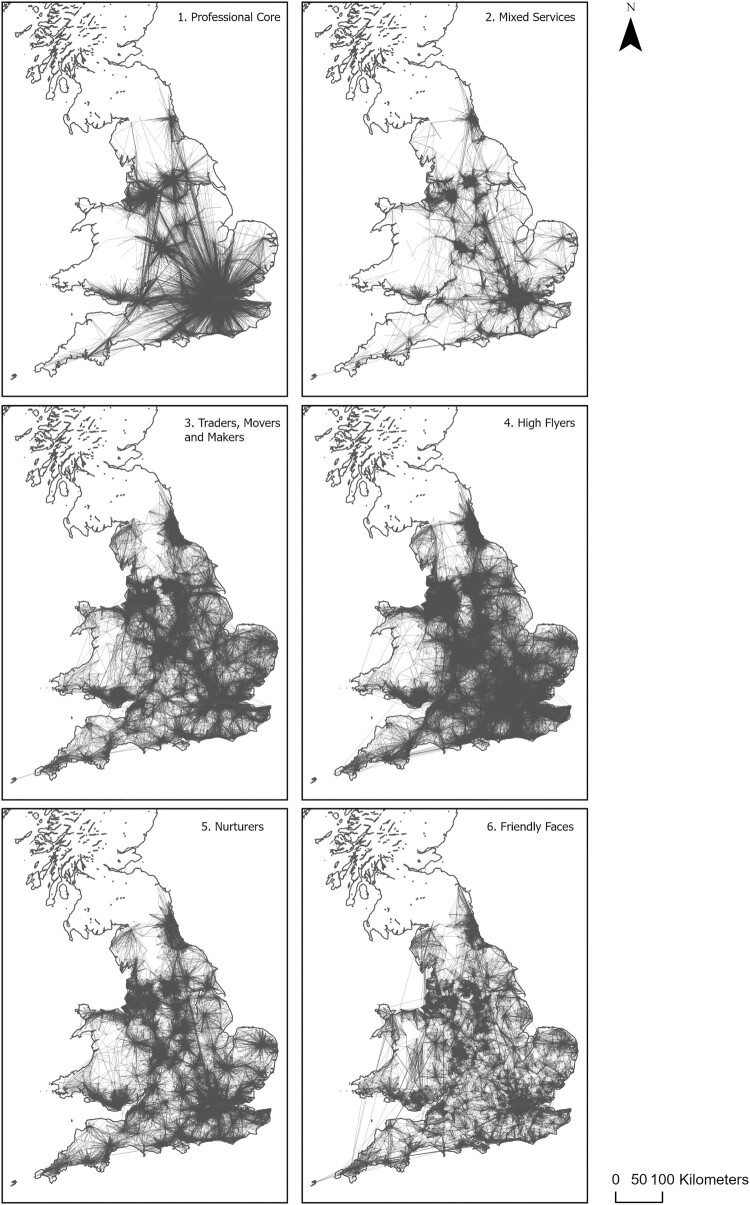
Commuting flows across England and Wales, segmented into distinct groups. Note: Patterns represent specific groups, showing the commuting connectivity between middle layer super output areas (MSOAs).

Mapping the patterns of the commuting flows underpinning each of the six groups reveals the structural variation that exists across the different configurations (Figure 1).^4^ Group 1 reflects a structure that is dominated by commuting into Greater London and the core cities of England and Wales. Group 2 reveals a similar structure but with a lower density of interactions. Groups 3–6 reveal a more extensive network of interactions with group 4 characterised by a dense network of flows.

Once the optimal number of clusters was determined, the next step involved profiling each cluster based on its underlying characteristics. To develop coherent cluster descriptions sensitive to indicator differences, we drew on metadata descriptions from the original census data, aligned to a review of the literature. A radial graph was created for each cluster (see Figure A2 in Appendix A in the supplemental data online) that were used to generate profiles for clusters that detailed the dominant characteristics of each group (Table 3). What is evident here is the variability in commuter profiles – some above, some below, and others around the grand mean for each variable. This variability underscores the complex patterns and structures underlying commuting within the LMAs.

**Table 3.** Geodemographic group profiles.

Group	Profile description
Professional core	This group has an above-average distribution of commuters employed fulltime across a range of socio-economic categories above the national average including higher managerial and administrative, higher, and lower professional, lower managerial and administrative and higher supervisory roles. The main associated industries include financial and insurance and professional and scientific and technical with a slightly above-average distribution in administrative and support services and public administration and defence. Occupational distributions above the national average include managerial, director and senior official roles, professional, associate professional and technical roles and administrative and secretarial roles near the national average. There are above-average levels of male commuters and above-average distribution of non-white commuters. Commuters in the 16–24- and 35–49-year age ranges are above the national average with levels of commuting by train also far above the national average. There is an above-average level of workers in the highest social grade category
Mixed services	This group has an above-average distribution of commuters employed part-time. Commuters in this group tend to be distributed across a range of socio-economic categories close to the national average, but are notably distributed above the national average in intermediate, lower supervisory, semi-routine and routine categories. The main associated industries include wholesale and retail trade and repair of motor vehicles, transport and storage, accommodation, and food and service activities alongside financial and insurance services, administrative and support services. Occupational distributions above the national average include administrative and secretarial, sales and customer services, and elementary occupations in supervisory, semi-routine or routine roles. The distribution of male and female commuters is comparable with the national average, but non-white commuters far exceed the mean. Commuters in the 16–24- and 35–49-year age ranges are above the national average with levels of commuting by train, walking, cycling and bus above the national average. There is an above-average level of workers in the middle to lowest social grade categories
Traders, movers and makers^a^	This group has an above-average distribution of commuters employed fulltime. Commuters in this group tend to be distributed above the national average in lower supervisory, semi-routine and routine socio-economic categories. The main associated industries include manufacturing, construction, wholesale and retail trade, and repair of motor vehicles, and transport and storage. The main associated industries tend to be manufacturing, construction, wholesale and retail trade, and repair of motor vehicles, and transport and storage. Occupational distributions above the national average overwhelmingly include skilled trades, process, plant and machine operations, and elementary occupations. The distribution of male commuters far exceeds the national average across the age ranges. Commuting by car and van exceeds the national average with all other modes of travel below the mean. There is an above-average level of workers in the lowest social grade category
High flyers^a^	This group has a higher-than-average distribution of commuters employed fulltime. Commuters in this group tend to be distributed above the national average across higher managerial and administrative, higher and lower professional, higher supervisory and lower supervisory socio-economic groups. The main associated industries include manufacturing, construction, financial and insurance industries, professional, scientific and technical activities, and public administration and defence. Occupational distributions above the national average include managerial, director and senior official roles, professional, associate professional and technical roles and administrative and secretarial roles. The distribution of male commuters far exceeds the national average and commuters in the 35–49- and 50–64-year age ranges are above the national average. Commuting by car far exceeds the national average with all other modes of travel below the mean. There is an above-average level of workers in the two highest social grade categories
Nurturers^a^	This group has an above-average distribution of commuters employed part-time (16–30 h) above the national average alongside a distribution of commuters employed part-time (≤ 15 h) or fulltime close to the national average. Commuters in this group tend to be distributed above the national average in the lower professional and technical socio-economic category and close to the mean in higher professional occupations, lower managerial and administrative, and intermediate groups. The main associated industries far above the national average include education and human health and social care, and public administration and defence near the mean. Occupational distributions above the national average include professional roles, and care, leisure and other service occupations. The distribution of female commuters far exceeds the national average and commuters in the 35–49- and 50–64-year age ranges are above the national average. Commuting by car far exceeds the national average with all other modes of travel below the mean. There is an above-average level of workers in the highest social grade category
Friendly faces^a^	This group has an above-average distribution of commuters employed part-time (16–0 h) above the national average. Commuters in this group tend to be distributed above the national average in the higher supervisory, intermediate lower supervisory, lower technical and semi-routine and routine socio-economic categories. The main associated industries above the national average include wholesale and retail trade and repair of motor vehicles, accommodation and food services, administrative and support services, education, and human health and social work. Occupational distributions above the national average include skilled trades, caring, leisure and other service occupations, sales and customer services, process, plant and machine operations and elementary roles. The distribution of female commuters far exceeds the national average and commuters in the 16–24- and 50–64-year age ranges are above the national average. Commuting by bus, cycling or walking exceeds the national average with all other modes of travel below the mean. There is an above-average level of workers in the two lowest social grade categories

Source: ^a^After Hincks et al. (2018).

## 5. DELINEATING GEODEMOGRAPHIC LMAs FOR ENGLAND AND WALES

In our LMA delineation exercise, we aim to identify LMAs for each geodemographic group using criteria to optimise LMA solutions (Table 4), where overall entropy is maximised across our chosen metrics, and where minimum self-containment and cohesion constraints are met (Table 5).

**Table 4.** Summary of evaluation metrics for labour market area (LMA) delineation.

Group	LMAs	Average area (km^2^)	Minimum supply (%)	Minimum demand (%)	Median self-containment (%)	Homogeneity (CV)	Balance (CV)
1. Professional core	12	12,847.9	66.2%	78.1%	87.9%	0.50	0.08
2. Mixed services	60	2569.5	63.4%	73.7%	92.7%	0.54	0.29
3. Traders, movers and makers	84	1835.4	60.4%	61.5%	81.7%	0.52	0.19
4. High flyers	51	3023.0	60.1%	60.0%	80.1%	0.53	0.37
5. Nurturers	69	2234.4	61.0%	64.0%	81.2%	0.56	0.29
6. Friendly faces	210	734.2	61.2%	67.1%	91.5%	0.48	0.06

Note: CV, coefficient of variation.

**Table 5.** Maximum entropy measures by group.

Group	LMAs (cohesion proxy)^a^	Entropy				
		Median supply self-containment	Median demand self-containment	Homogeneity	Balance	Overall maximum entropy
1. Professional core	12	0.84	0.92	0.73	0.08	1.20
2. Mixed services	60	0.91	0.92	0.97	0.29	1.31
3. Traders, movers and makers	84	0.77	0.79	0.55	0.21	1.29
4. High flyers	51	0.81	0.82	0.56	0.21	1.28
5. Nurturers	69	0.80	0.82	0.56	0.29	1.32
6. Friendly faces	210	0.92	0.91	0.48	0.06	1.15

Note: ^a^The metric is not included in the overall maximum entropy calculation.

Trends in individual metrics are informative. Higher median self-containment values (both supply and demand) and increased homogeneity are generally associated with fewer LMAs. This is consistent with larger regions containing more commuting flows and having more uniform sizes. The balance metric exhibits wider variation, reflecting the complexity of optimising job–resident ratios influenced by factors beyond the number of LMAs.

Overall entropy values range from 1.15 to 1.32, indicating variability across the solutions. Interestingly, entropy values do not exhibit a strict monotonic relationship with the number of LMAs, which is considered here as a proxy for cohesion. The solution with the highest number of LMAs, friendly faces, exhibits the lowest entropy at 1.15, indicating the lowest randomness and highest uniformity in the distribution of the metrics. Conversely, the nurturers solution, with a relatively high number of LMAs (69), records the highest entropy at 1.32, suggesting greater randomness and variability.

Professional core has the fewest LMAs (12) and an entropy of 1.20, indicating a more uniform distribution of metrics, which may foster stronger internal cohesion. Mixed services (60) and traders, movers and makers (84) have entropies of 1.31 and 1.29, respectively, indicating relatively high variability. Similarly, high flyers (51 LMAs) has an entropy of 1.28, showing significant variability. Overall, the results reflect the complexity of balancing different metrics when optimising the number of LMAs. While a higher number of LMAs can sometimes lead to fragmented interactions and higher entropy, fewer LMAs can foster stronger internal cohesion and lower entropy.

**Figure 2.**
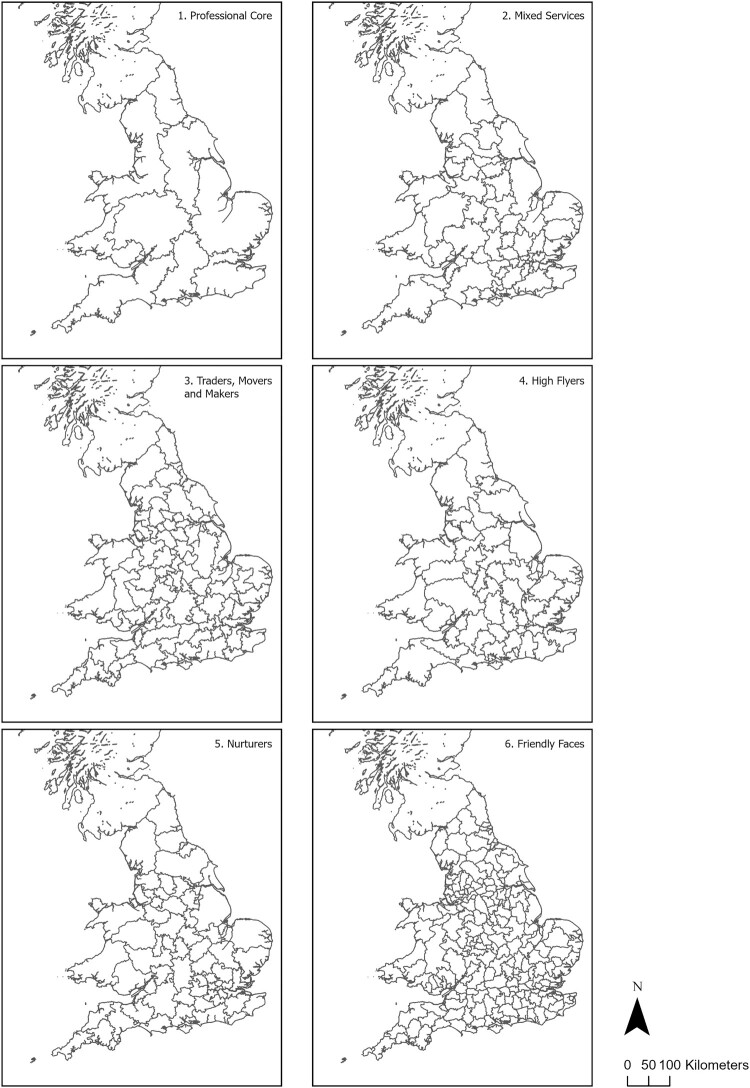
Geodemographic labour market areas, segmented into distinct regions.

The final LMA configuration is mapped in Figure 2. What emerges for professional core is a suite of LMAs that is predominantly ‘regional’ in structure, where core metropolitan areas, and especially Greater London, attract significant inflows of workers from across an extensive geographical area. In contrast, the friendly faces group is more geographically concentrated, reflecting a much more localised commuting structure underpinned by modes of travel that include bus, cycling or walking. In the derivation of the 2011 alternative TTWAs, the ONS (2016) observed that fewer LMAs were typically associated with larger LMAs and generally longer distance commuting, while higher numbers of LMAs were typically associated with smaller LMAs underpinned by shorter distance commuting, higher concentrations of local commuting patterns and higher proportions of routine, semi-routine and intermediate workers (Casado-Díaz, 2000; Coombes et al., 1988; ONS, 2016).

Although only a measure of the average straight-line distance between MSOAs calculated between-centroids (Table 6), the measure of the average distance commute highlights that professional core, traders, movers and makers and high flyers all exceed the national median distance commute of 10.5 km, while commuting dispersion – measured through the standard deviation (SD) (Hincks et al., 2018) – is relatively consistent across all groups, except for professional core, which records the highest mean and median commuting distances of any of the groups along with the highest SD. This pattern is consistent with managerial and professional socio-economic groups and a mode of travel where train far exceeds the national modal average (Casado-Díaz, 2000; Coombes et al., 1988; Farmer & Fotheringham, 2011; Green et al., 1986; Hincks et al., 2018; Hincks & Wong, 2010). Extending the focus to consider commuting connections, traders, movers and makers, high flyers and friendly faces record flows more than the national average. In terms of workforce population, mixed services and friendly faces rank top of all groups, both exceeding the national average of 16.7%. Professional core is ranked lowest on the number of connections and second lowest behind nurturers in terms of workforce population (Table 7).

**Table 6.** Measures of commuting distance by group.

Group	Median commutingdistance (km)	SD
1. Professional core	16.8	27.8
2. Mixed services	6.1	12.6
3. Traders, movers and makers	11.7	14.4
4. High flyers	16.3	14.4
5. Nurturers	10.3	13.1
6. Friendly faces	5.1	14.5
		
England and Wales	10.5	17.2

**Table 7.** Measures of the structure of commuting by group.

Group	Connections by group		Workforce by group	
	Connections	Total connections (%)	Workers	Total workers (%)
1. Professional core	63,393	12.3%	1,818,338	9.9%
2. Mixed services	74,207	14.4%	3,309,488	18.0%
3. Traders, movers and makers	92,488	18.0%	2,670,606	14.5%
4. High flyers	103,932	20.2%	2,096,951	11.4%
5. Nurturers	80,082	15.6%	1,547,136	8.4%
6. Friendly faces	99,790	19.4%	6,959,314	37.8%
				
Mean	85,648	16.7%	3,066,972	16.7%
Total	513,892	100.0%	18,401,833	16.7%

**Figure 3.**
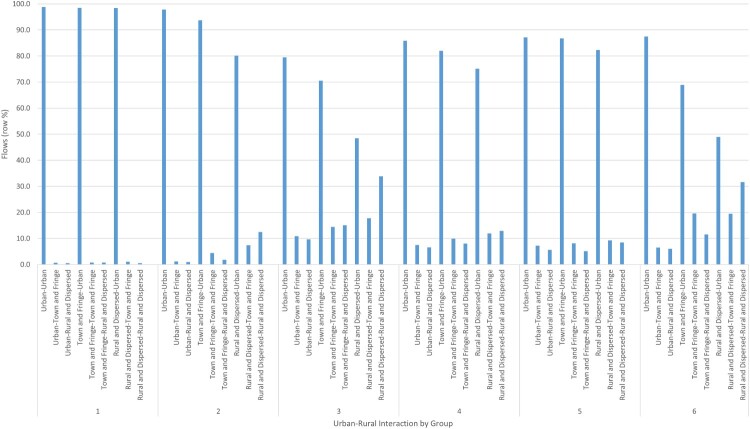
Commuting flows by settlement type from urban, suburban, and rural areas. Note: Different line heights represent varying flow intensities, labelled with settlement type-specific groupings: (1) professional core; (2) mixed services; (3) traders, movers and makers; (4) high flyers; (5) nurturers; and (6) friendly faces.

In delineating LMAs, it has been noted elsewhere that urban–rural differences in commuting patterns and behaviours are likely to condition the size and geography of defined regions (Casado-Díaz, 2000; Coombes et al., 1988; Farmer & Fotheringham, 2011; Green et al., 1986; Hincks & Wong, 2010). Drawing on the 2011 Urban–Rural Classification of Small Area Geographies,^5^ it is evident that while urban-orientated commuting patterns feature prominently across all groups, there are also notable variations and contrasts in the geographies of interactions beyond the urban (Figure 3). As something of an outlier, professional core is largely dominated by commuting flows concentrated towards core urban areas and Greater London.

It is widely recognised that the segmentation of commuting flows for use in the delineation of subgroup LMAs can result in a sparseness of flows that has the potential to undermine the interpretability and robustness of regionalisation solutions (Casado-Díaz, 2000; Coombes et al., 1988; Green et al., 1986). While the proportional sample of flows underpinning each group of our geodemographic classification is not out of step with those employed in other studies (e.g., Coombes et al., 1988; Casado-Díaz, 2000; Farmer & Fortheringham, 2011), our approach to segmenting commuter flows based on different demographic, socio-economic and modal characteristics remains susceptible to issues of sample size, especially in relation to rural MSOAs where commuting interactions tend to be reduced when compared with urban MSOAs.

Nevertheless, the extent of variation in the distribution of connections, workforce size, commuting distance, and dispersion is indicative of the effect of demographic and socio-economic characteristics on commuting patterns and behaviours. Likewise, urban–rural dynamics are understood to accentuate variations within and between metropolitan and non-metropolitan LMA structures (Green et al., 1986) that are reflected in differences in LMA geographies (e.g., sizes and shapes).

## 6. GEODEMOGRAPHIC LMAs, PRODUCTIVITY AND UNEVEN SPATIAL DEVELOPMENT IN ENGLAND AND WALES

In this section, we use our geodemographic LMAs to explore patterns of uneven spatial development in England and Wales. This approach is valuable as TTWAs and other functional economic geographies have been employed in studies of spatial inequality (e.g., Coombes, 2014; Hincks & Wong, 2010; Jones, 2017). Productivity is a widely adopted indicator of economic performance and disparities in productivity across subnational areas of the UK are striking, both in absolute terms and by international standards. UK regional inequalities have a long history, stretching back more than a century (McCann, 2016), where regional inequalities have widened between the north and south of the UK, characterised by falling output to the national economy and lower incomes and employment opportunities in northern cities and regions (Martin, 2015, p. 241).^6^

Zymek and Jones (2020)^7^ contend that regional productivity is influenced by ‘place fundamentals’, such as geography, culture, governance and infrastructure; ‘agglomeration’ of economic activities; and the ‘sorting’ or workers based on residential and workplace locations and skill profiles that shape the ‘industry mix’ of a place. In this section, we aim to shed light on uneven spatial development in England and Wales, focusing on infrastructure and agglomeration disparities alongside the effects associated with the ‘sorting’ of workers based on residential, workplace and socio-demographic characteristics. These are represented through the group configuration of LMAs defined above, while we also compare our LMAs to other subnational economic geographies.^8^

Our entry point here is the now stylised agglomeration-based economic theory, where we examine the relationships between settlement size, productivity, and infrastructure efficiencies. Under the assumptions of agglomeration, larger settlement areas lead to higher economic productivity and infrastructural efficiencies due to increased potential for population mixing and lower transport costs. This has led to policy arguments advocating the development of polycentric regions through better intercity transport infrastructure, such as the Dutch Randstad and the German Rhine-Ruhr metropolitan regions.

We employ the theory of settlement scaling to consider the claims of agglomeration benefits. In doing so, scaling theory allows us to measure – in agglomeration terms – the characteristics of settlements as population scaling functions, drawing on analogous allometric relationships observed in the growth and size of organisms (Bettencourt et al., 2007). Recent analyses purport to demonstrate population dependence of various settlement characteristics, from economic output and crime to prevalence of viral diseases and road lengths (Gomez-Lievano et al., 2016) and suggest allometric power laws and the presence of ‘universal features’ among settlements (Bettencourt & West, 2010). The generic formulation for these power law relationships is represented in a log transformed form as:

(11) $$\ln\{F(N)\}=\ln(F_{0})+\mathrm{\beta ln}N\;\;\;\;\;\;\;\;\;\;\;\;\;\;\;$$

where *F* represents any chosen settlement indicator (e.g., economic output, urbanised area, CO_2_ emissions), *F*_0_ is the baseline prevalence of the indicator, *N* is the settlement population count, and *β* is the scaling exponent determining the growth regime. Bettencourt (2013) shows, under the four assumptions:

- The average aggregate socio-economic product is a linear function of the sum of all local interactions.
- Settlement population is mixing uniformly, and individuals have the minimum resources that are needed to travel and experience the place fully (Glaeser & Kohlhase, 2003).
- Individual baseline production is bounded and is not a function of settlement size (Szüle et al., 2014).
- Infrastructure is embedded as a hierarchical network that keeps all individuals connected through its incremental and decentralised growth (Samaniego & Moses, 2008).

Here infrastructural indicators such as built-up area and road length grow sublinearly with population (*β*_An_ ≈ 0.56) while indicators of productivity, such as gross domestic product (GDP) exhibit superlinear growth (*β*_Y_ ≈ 0.76). To this end, a balance is thought to exist between economic output and associated congestion costs as a function of interactions between productivity and density (Bettencourt et al., 2007). It is notable, however, that agglomeration benefits disappear when assumptions (b) and (d) are violated. As such, economic exponents closer to one could imply that people are not able to mix adequately due to densities and/or mobility infrastructure that has not developed as anticipated, whether as a result of poor provision or artificially because of choice of boundaries not capturing full LMAs (e.g., administrative boundaries).

Against this backdrop, Arbabi et al. (2019; 2020) have shown that productivity disparities in the North of England coincide with poorer intra-city mobility across various density-based geographies. It is widely accepted that administrative boundaries (e.g., local authority) lack sensitivity to functional interactions where measures of productivity, population size and mixing, and infrastructural capacities are constrained by political rather than functional processes. Arbabi et al. (2019) found that applying scaling within a framework of functional geographies, such as TTWAs, led to a close alignment in estimates of productivity – measured using gross value added (GVA) – and settlement land-area scaling exponents, corresponding to those prescribed by the original settlement scaling model. This alignment largely upheld the mixing population assumption. Building on their analysis, we examine agglomeration and mobility effects on GVA through the lens of geodemographic LMAs and comparators in the form of TTWAs, functional urban areas, and local authority boundaries. This approach allows us to compare settlement scaling and the assumptions outlined above (a–d) across different administrative and functional geographies, where variations in agglomeration, population size, mixing and infrastructural capacities exist.

**Figure 4.**
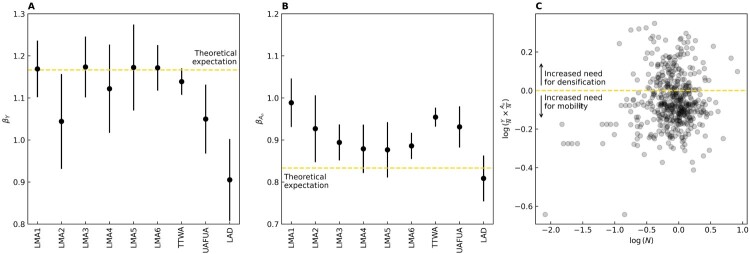
Elasticity of returns to scale across different geodemographic boundaries: ordinary least squares (OLS) estimates of scaling exponents *β* for (a) economic output, (b) the extent of the urbanised area, and (c) the balance between economic output and infrastructural efficiencies as captured by the interaction of productivity and density for all geographies. Note: Values of mean-normalised distribution reflect the increased need for densification or mobility by area. LMA1 (professional core), LMA2 (mixed services), LMA3 (traders, movers and makers), LMA4 (high flyers), LMA5 (nurturers), LMA6 (friendly faces), TTWA (travel-to-work area), UAFUA (unitary authority functional urban area) and LAD (local authority district).

Figure 4 summarises the estimated scaling exponents for the geodemographic LMAs, which we compare with other economy-wide boundary systems, including TTWAs. What is clear from Figure 4a in relation to the different boundary systems we analysed is that the economic agglomeration effects are generally strong and close to the theoretical expectations (dotted line), except for weaker effects in unitary authority functional urban areas (UAFUAs) and the geodemographic LMA2, mixed services group. Moreover, the urbanised area exponents tend to be larger than expected, represented by the exponents exceeding the theoretical expectation for all functional areas, but falling below the theoretical expectation for local authority boundaries (Figure 4b). Under a scaling lens, this can be interpreted as an underdevelopment of local means of mobility and access necessitating larger conurbations for similar economic agglomeration effects. Figure 4c shows productivity and density interactions, which should be independent of population under the four assumptions above. Variations from the baseline could indicate infrastructural disparities, where the positive trend reflects economically successful cities that have grown in extent and could benefit from densification. The negative trend, on the other hand, comprise cities that struggle to meet their assumed population potential because of poor internal mobility and mixing.

At this point we turn to reflect on the trends in the new geodemographic-based LMAs. It is worth noting here that the LMAs in each of the groups were derived based on a subgroup population of commuters that are expected to be more similar than dissimilar in their underlying socio-economic and demographic characteristics (Hincks et al., 2018). Here we draw on Dorling’s (2010) north–south divide, stretching from the Wash in the east to the Severn Estuary in the south-west, as exemplifying a regional geography of spatial inequalities in the England and Wales.^9^ In doing so, we can compare agglomeration-based metrics and spatial differences across different boundary systems, but crucially between geodemographic LMAs above and below the north–south divide that are formed from commuters of similar characteristics.

**Figure 5.**
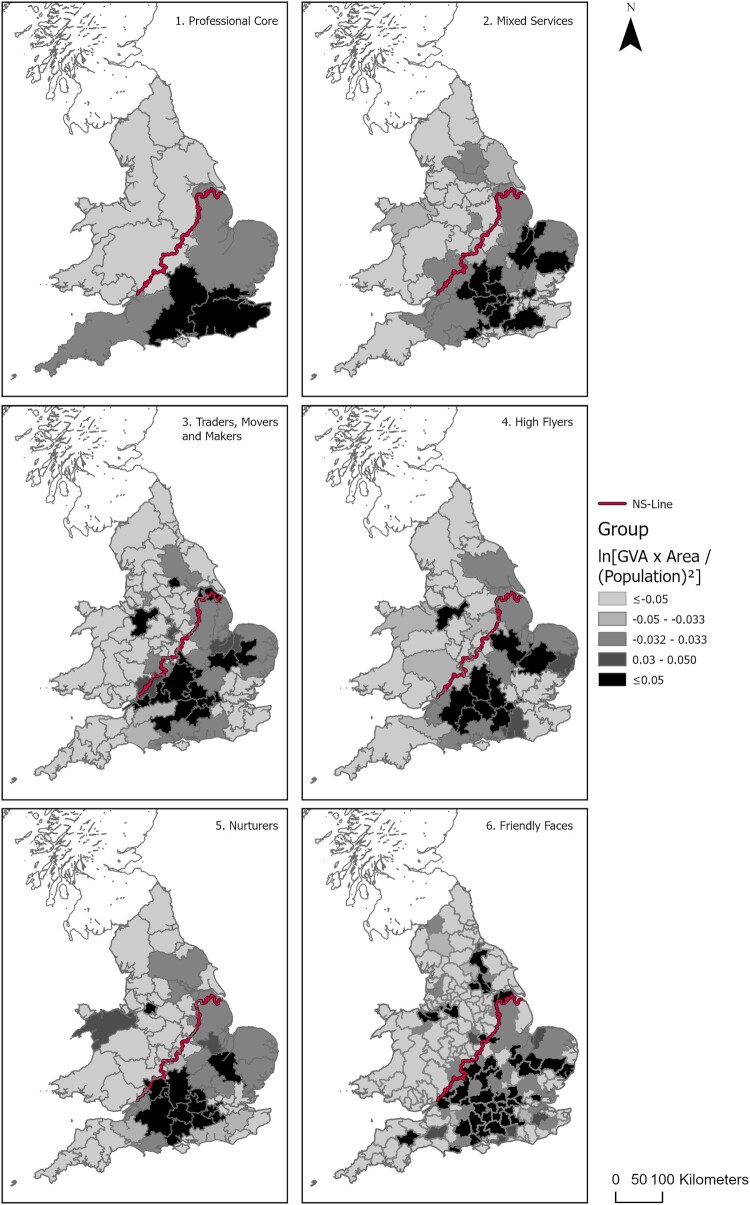
Densification and mobility across geodemographic labour market areas (LMAs). Note: Darker areas indicate increased benefits from densification (positive log(*YN* × *A* *nN*) values in (c), while lighter areas indicate increased benefits from better internal mobility.

Figure 5 shows the interaction of productivity and density, revealing variable tendencies for densification or mobility compared with the Wash–Severn representation of the north–south divide, across geodemographic regions. The darker grey indicates increased benefits from densification measured as a positive log of the values in Figure 4c, while the lighter grey areas are suggestive of increased benefits that might be derived from improvements in internal mobility.

What is revealed is that LMAs with subgroup populations that share similar socio-economic and demographic characteristics perform differently north and south of the divide. This is especially pronounced in relation to the professional core, but is a consistent feature across all groups. While infrastructural challenges are evident across the south-west region, generally there is a greater tendency towards densification below the Wash–Severn line than there is above it. Here productivity differences likely stem from historical and sustained unevenness in infrastructure provision, along with other structural factors to which boosterist agglomeration arguments often struggle to respond (Haughton et al., 2014; Hincks et al., 2017). For decades, UK infrastructure policy and investment have been predominantly London and south-east-centric (Martin et al., 2016; McCann, 2016; O’Brien et al., 2019), doing little to tackle a profoundly imbalanced and inequitable national space economy (Haughton et al., 2014, p. 266; Martin et al., 2022).

## 7. DISCUSSION AND CONCLUSIONS

This paper offers on a novel contribution to debates on the delineation and application of LMAs in economic planning and regional studies through a focus on tackling a long-standing question: how do the commuting behaviours of different workforce groups affect the geography, structure, and productivity of functional LMAs? (Karlsson & Olsson, 2006; ONS, 2016; Van der Laan & Schalke, 2001).

Our analysis reveals that commuting behaviours of different workforce groups have notable implications for the geography, structure, and productivity of functional LMAs. In the first phase of the analysis, we define for the first time, geodemographic-based subgroup LMAs for England and Wales, integrating a new geodemographic classification of commuting flows within a functional regionalisation framework. Across the six groups, we delineated 486 LMAs, informed by measures of autonomy, homogeneity, balance and cohesion, reflecting extensive variation in commuting behaviours that lead to variations in the number of LMAs defined for each group, ranging from 12 in the professional core group to 210 in friendly faces. The median number of LMAs across all groups is 65, with SD = 67.7. LMAs in professional core cover the largest area on average (12,847 km^2^), while the friendly faces LMAs are more compact on average (734 km^2^). Professional core is characterised by a predominantly ‘regional’ LMA structure, with core metropolitan areas attracting workers from an extensive geographical area, drawing on high concentrations of professional and managerial workers. In contrast, friendly faces has a more localised commuting structure supported by bus, cycling or walking.

The extent of variation in commuting structures is further demonstrated through analysis of commuting distances. The professional core group, consisting of managerial and professional workers, exhibits the highest mean and median commuting distances, along with the highest SD. In contrast, traders, movers and makers and high flyers exceed the national median distance commute of 10.5 km. Commuting dispersion, measured through SD, was relatively consistent across all groups, except for professional core, which records the highest mean and median commuting distances of any of the groups along with the highest SD. This trend is consistent with the findings of other studies where the prevalence of more distant and more dispersed commuting patterns of higher status groups creates fewer but larger LMAs compared with those patterns comprised of semi-routine or routine workers or greater concentrations of active modes of travel (Casado-Díaz, 2000; Coombes et al., 1988; ONS, 2016).

Urban–rural differences were also found to play a role in shaping the geography of commuting interactions and, consequently, the structure of LMAs. While urban-oriented commuting patterns are prevalent across all groups, variations emerge beyond urban areas. The outlier was professional core, which is dominated by commuting flows directed towards core urban areas and Greater London. In the post-COVID context, remote and flexible homeworking practices have altered commuting patterns (ONS, 2022), reflected in modal shifts (Magriço et al., 2023) and socio-demographic segmentation (Richards et al., 2024). Analysing commuting patterns and delineating segmented LMAs that are sensitive to commuter characteristics and urban–rural contrasts could offer an approach to understanding evolving commuting and spatial labour market structures across increasingly flexible home–work interactions.

Having delineated the new suite of LMAs, we then draw on settlement scaling theory to explore the strength of economic and infrastructure agglomeration effects in England and Wales using the new geodemographic LMAs as our spatial units. In doing so, we offer a contribution to a broader discussion concerning economic productivity and regional spatial inequalities in England and Wales (Arbabi et al., 2019; Martin et al., 2016; McCann, 2016; O’Brien et al., 2019). The findings indicate strong and generally expected economic agglomeration effects with the exponents for urban areas being larger than anticipated, suggesting an underdevelopment of local mobility and access that necessitate even larger urban areas for comparable economic agglomeration effects.

We then extend the analysis consider trends within the new geodemographic-based LMAs. The LMAs are defined based on subgroup populations of commuters expected to share similar socio-economic and demographic characteristics. Drawing on Dorling’s notional north–south divide to reflect a proxy for regional spatial inequalities, we compare agglomeration metrics and their variations above and below the north–south divide. Significantly, we found variations in performance of LMAs, characterised by subgroup populations sharing similar characteristics, north and south of the divide. Our analysis suggests that differences in productivity may be influenced by differences in infrastructure (alongside other structural factors), reflecting historical and sustained unevenness in infrastructure provision in the UK (Martin et al., 2016; McCann, 2016; O’Brien et al., 2019).

Against these findings, there are possibilities to extend the research undertaken here. The LMAs we defined were conditioned by the conceptual and methodological decisions taken to create the geodemographic classification. It is also the case that segmenting commuting flows for delineating subgroup LMAs can lead to sparse flows that impact the interpretability and robustness of regionalisation solutions. While the proportional sample of flows in each subgroup aligns with other studies, the approach to segmenting commuter flows based on demographic, socio-economic and modal characteristics remains susceptible to sample size issues, especially in rural areas (Coombes et al., 1988; Casado-Díaz, 2000; Farmer & Fortheringham, 2011).

As a hierarchical clustering algorithm, Intramax is effective but has certain limitations. It prioritises the merging of base units with high interactions without adapting based on previous mergers. This can result in large, well-contained regions alongside smaller, poorly self-contained ones (Martínez-Bernabeu et al., 2020). In contrast, the TTWA method addresses these deficiencies by first merging regions with poorer characteristics, ensuring more homogenous regions in terms of self-containment and size. Although the TTWA algorithm achieves lower self-containment levels than Intramax, its regions are often more cohesive and evenly sized (Martínez-Bernabeu et al., 2020). Extending the focus of our work to derive UK-wide geodemographic classifications and TTWA geographies would offer a further novel contribution in this area.

Martínez-Bernabeu et al. (2020) also suggest that their cohesion interaction index could provide a more reliable measure of cohesion than the number of LMAs. This metric could be integrated into future studies, alongside a comparison of the Intramax and TTWA frameworks using a geodemographic classification of origin–destination flows. Finally, the approach outlined here could be adapted for use in other countries and contexts where disaggregated origin–destination flow data are available, aiding in the delineation of subgroup geodemographic LMAs.

## Supplementary Material

Supplemental Material

## NOTES

1. Alternative Travel to Work Areas (arcgis.com).

2. MSOAs are census units in England and Wales, totalling 7201 with population ranges of 5000–15,000 (2011-based).

3. Notably, the second ranked cluster configuration was a nine-cluster solution, as identified by Hincks et al. (2018), in their original classification of commuting flows for England and Wales.

4. For higher resolution versions of the maps, see Appendix A in the supplemental data online.

5. The 2011 Urban Rural Classification was developed using Census of Population data (Rural Urban Classification – GOV.UK; www.gov.uk). It defines areas as rural if they fall outside of settlements with more than 10,000 resident population. For small areas geographies – including MSOAs – the classification assigns areas to one of four urban or six rural categories: Rural: hamlets and isolated dwellings; Rural: hamlets and isolated dwellings in a sparse setting; Rural: village; Rural: village in a sparse setting; Rural: town and fringe; Rural: town and fringe in a sparse setting; Urban: city and town; Urban: city and town in a sparse setting; Urban: minor conurbation; and Urban: major conurbation. For present purposes, we combine these categories into three dimensions: urban, town and fringe; and rural and dispersed.

6. The UK2070 Commission – an independent inquiry into city and regional inequalities in the UK – was established to promote interventions to address persistent inequalities between the UK cities and regions.

7. The Industrial Strategy Council is an independent non-statutory advisory group to the UK government, established in 2018, and charged with providing impartial evaluation and advice on progress towards the delivery of the UK’s Industrial Strategy.

8. We have used the 2011 TTWA, the 2016 Urban Audit’s functional urban area (UAFUA), and the 2022 local authority district (LAD) boundaries for this comparison, where TTWAs and UAFUAs are taken to represent coherent urban boundaries where we expect to see stronger agglomeration effects and LADs comprise arbitrary subdivisions of areas causing diminishing observed agglomeration due to the modifiable areal unit problem (MAUP).

9. While the ‘Severn-Wash’ line is one representation of a spatial divide in the UK that might appear relatively simplistic, McCann (2016, p. 18) notes that:
In economic terms the UK is characterised by an incredibly strong core–periphery spatial structure whereby the ‘core’ was traditionally defined as the regions located to the south of an imaginary line drawn between the River Severn and The Wash and the periphery being those regions located to the north of this line. In economic and wellbeing terms this basic divide now holds stronger than ever, except for the fact that an additional imaginary line demarcating the economic ‘core’ from the ‘periphery’ nowadays also exists, which is the line describing the border between northern English regions and Scotland.
