# Supplementary material for: Building geodemographic regions: commuting, productivity and uneven spatial development in England and Wales
Source: Reg Stud. 2025 May 6;59(1):2485132. doi: 10.1080/00343404.2025.2485132 (PMC12306677; doi:10.1080/00343404.2025.2485132)
Supplement: Supplemental Material [file CRES_A_2485132_SM3079.pdf]

APPENDIX A

A1: Commuting Flows Segmented by Groups

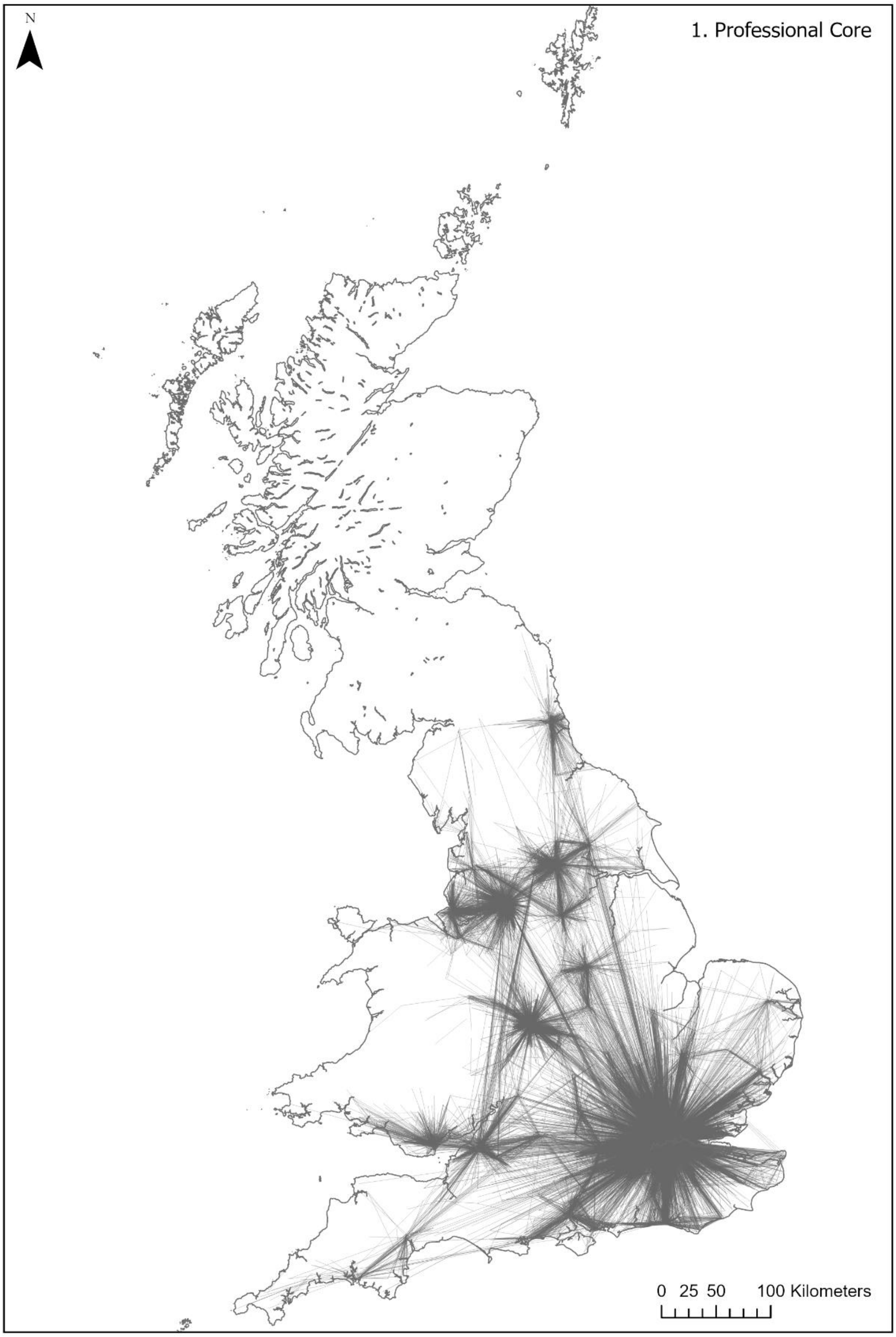

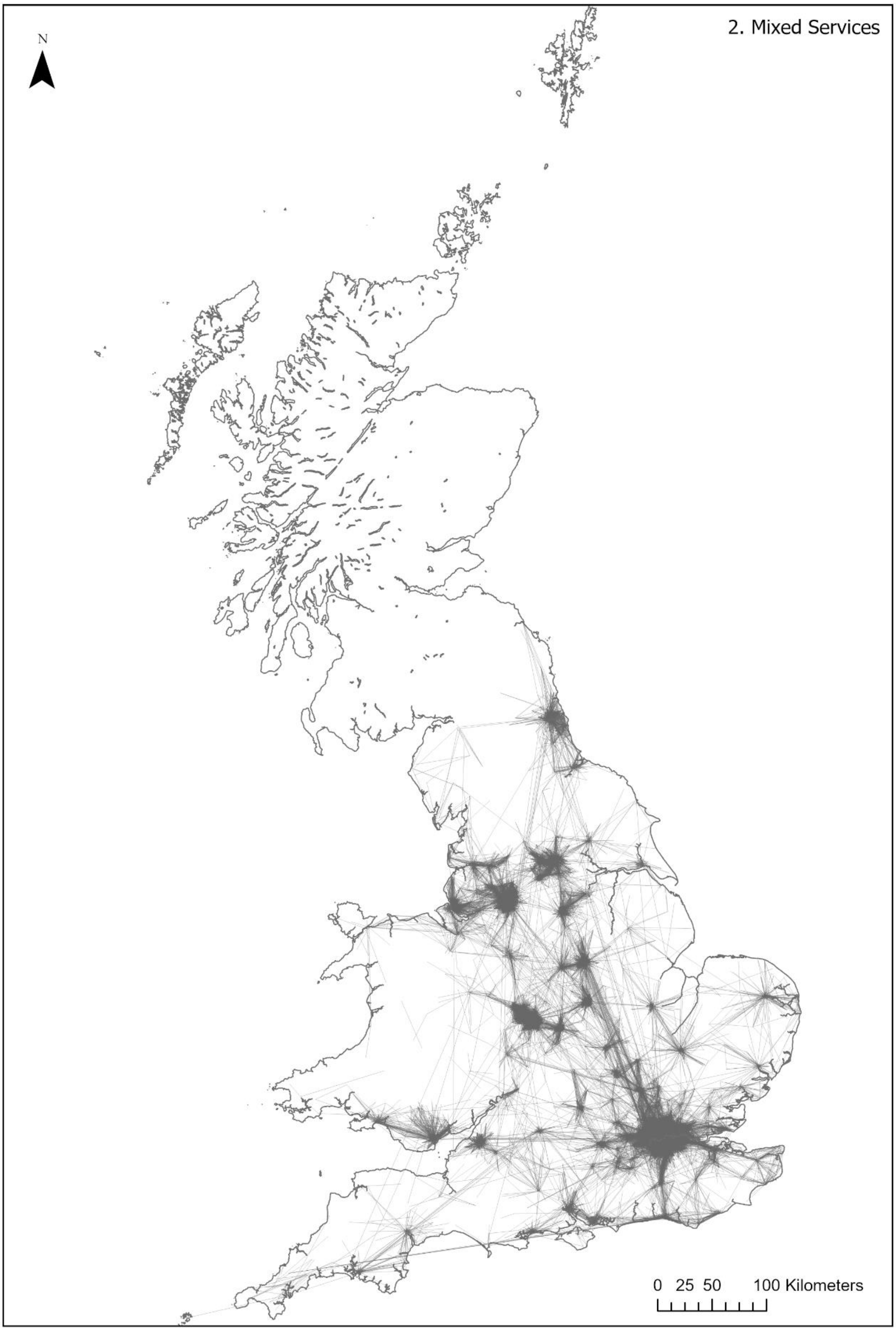
2. Mixed Services
N
0 25 50 100 Kilometers

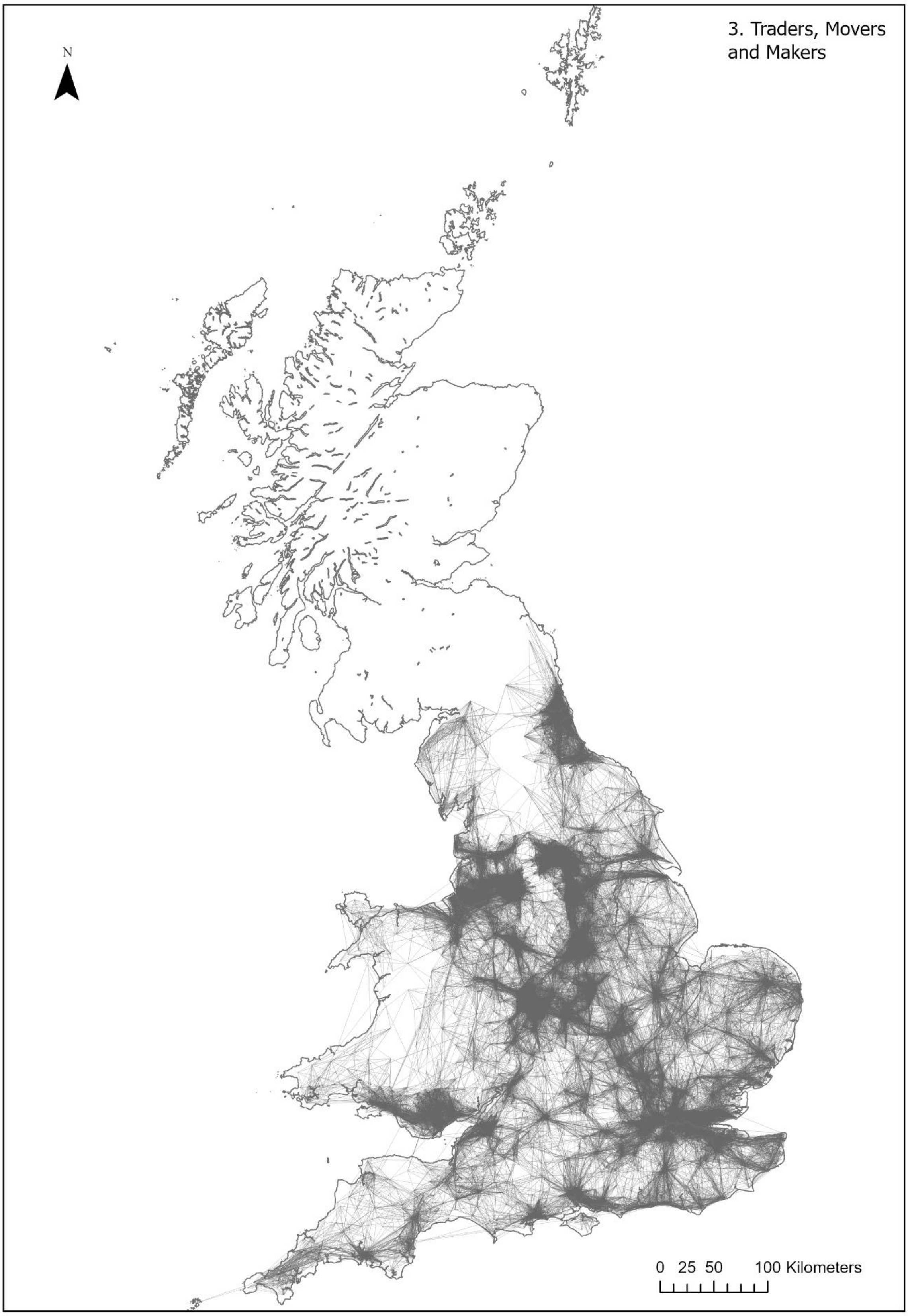

3. Traders, Movers and Makers
N
0 25 50 100 Kilometers

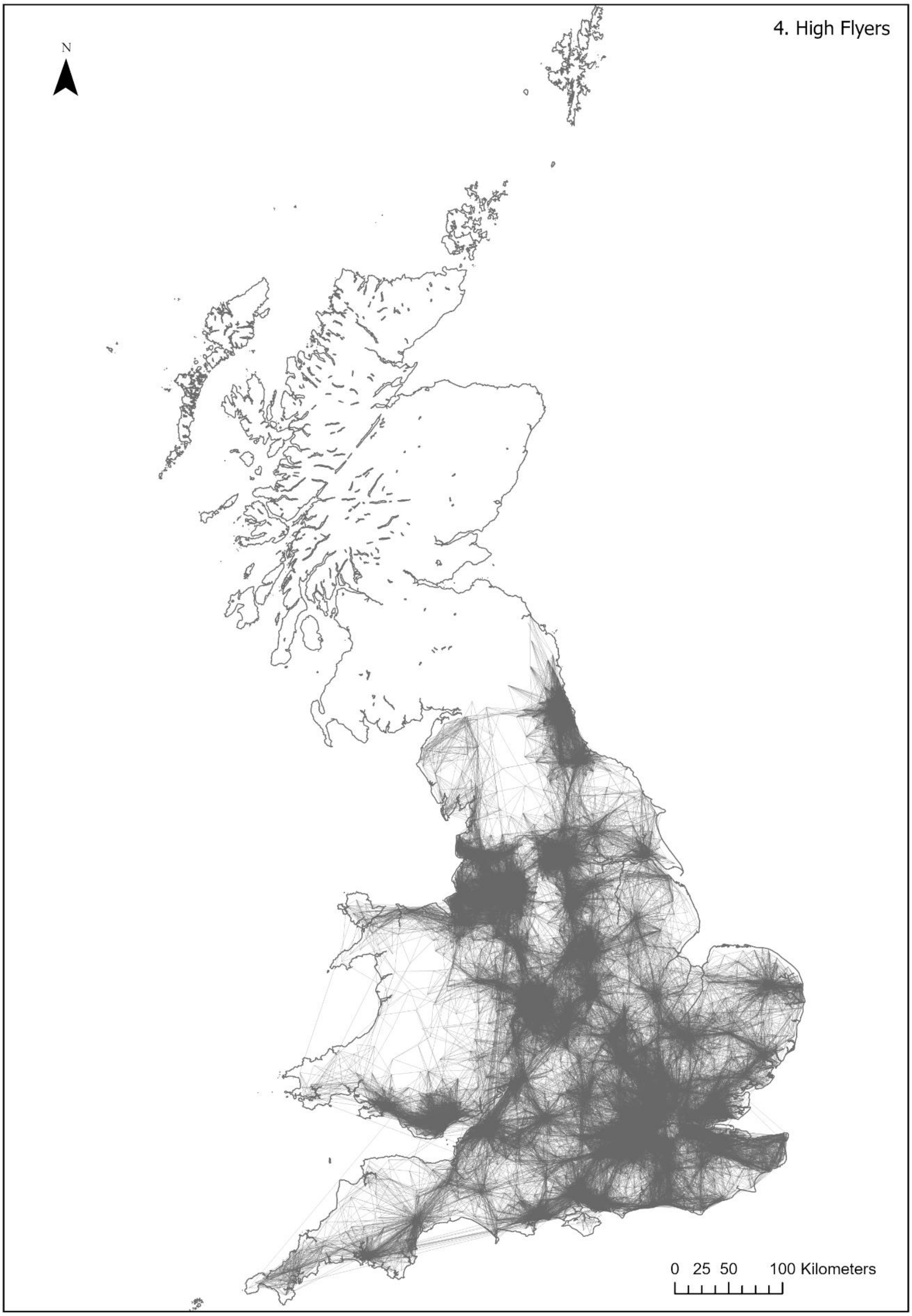
4. High Flyers
N
0 25 50 100 Kilometers

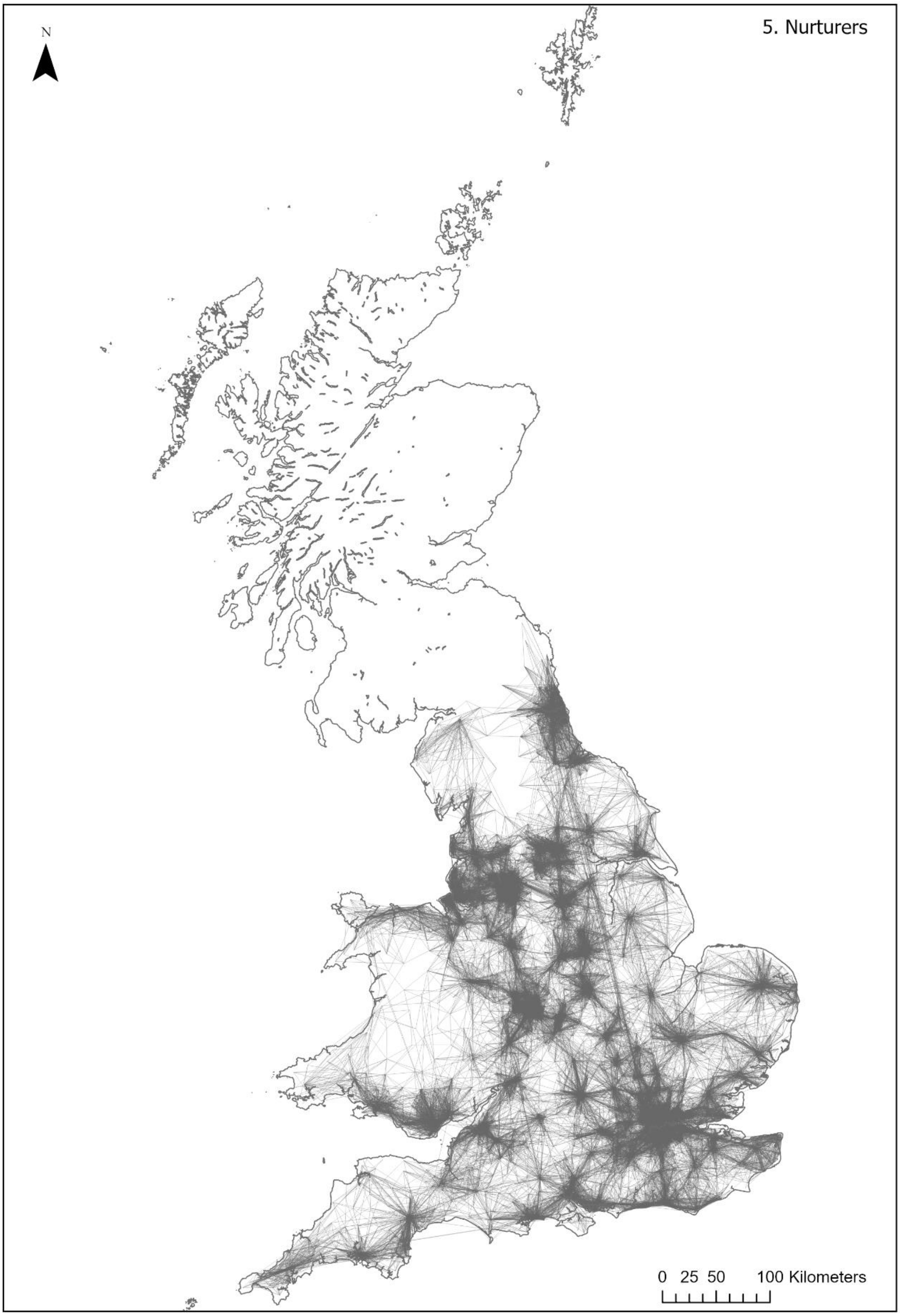

5. Nurturers
N
0 25 50 100 Kilometers

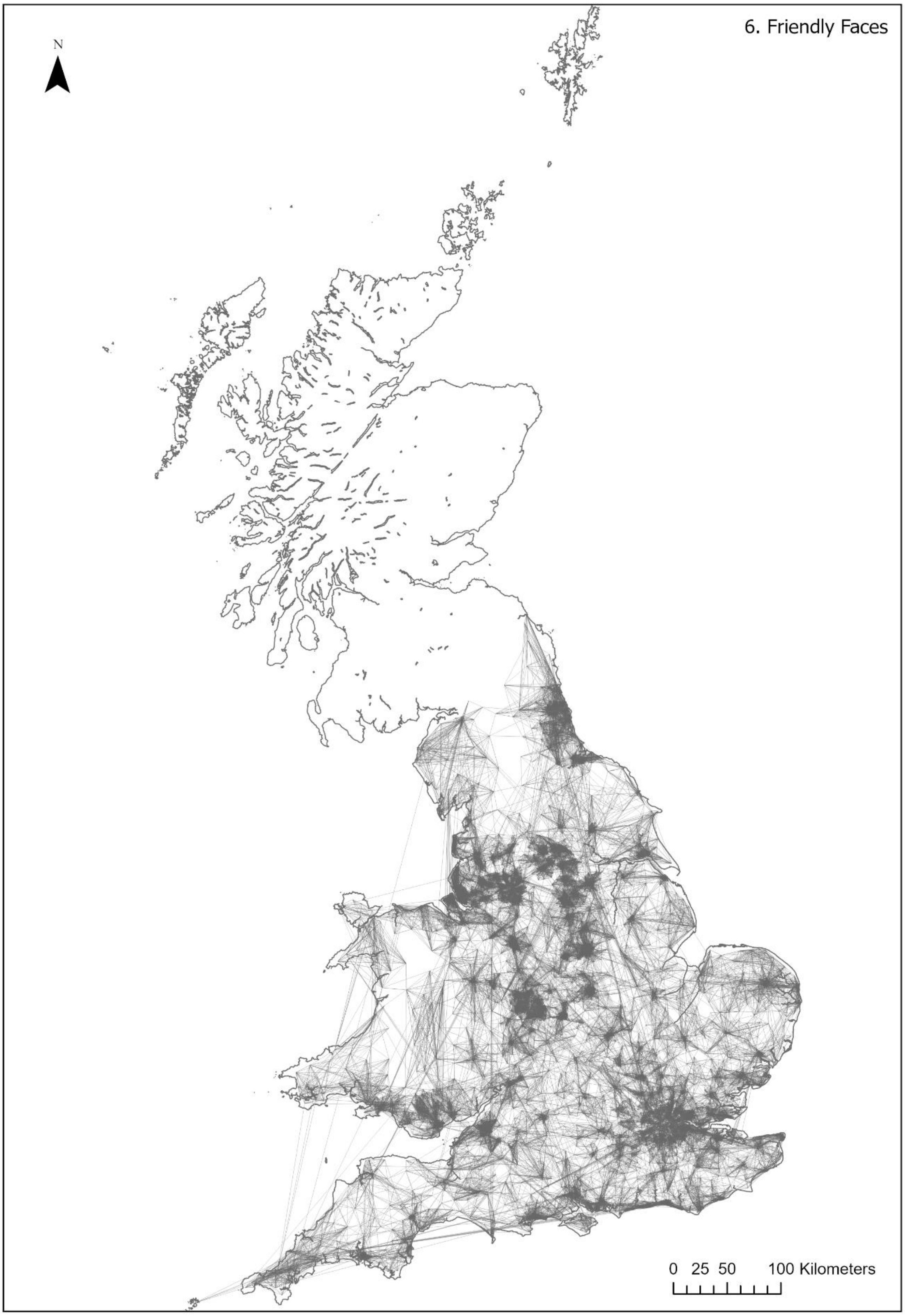
6. Friendly Faces
N
0 25 50 100 Kilometers

A2: Geodemographic Group Radials

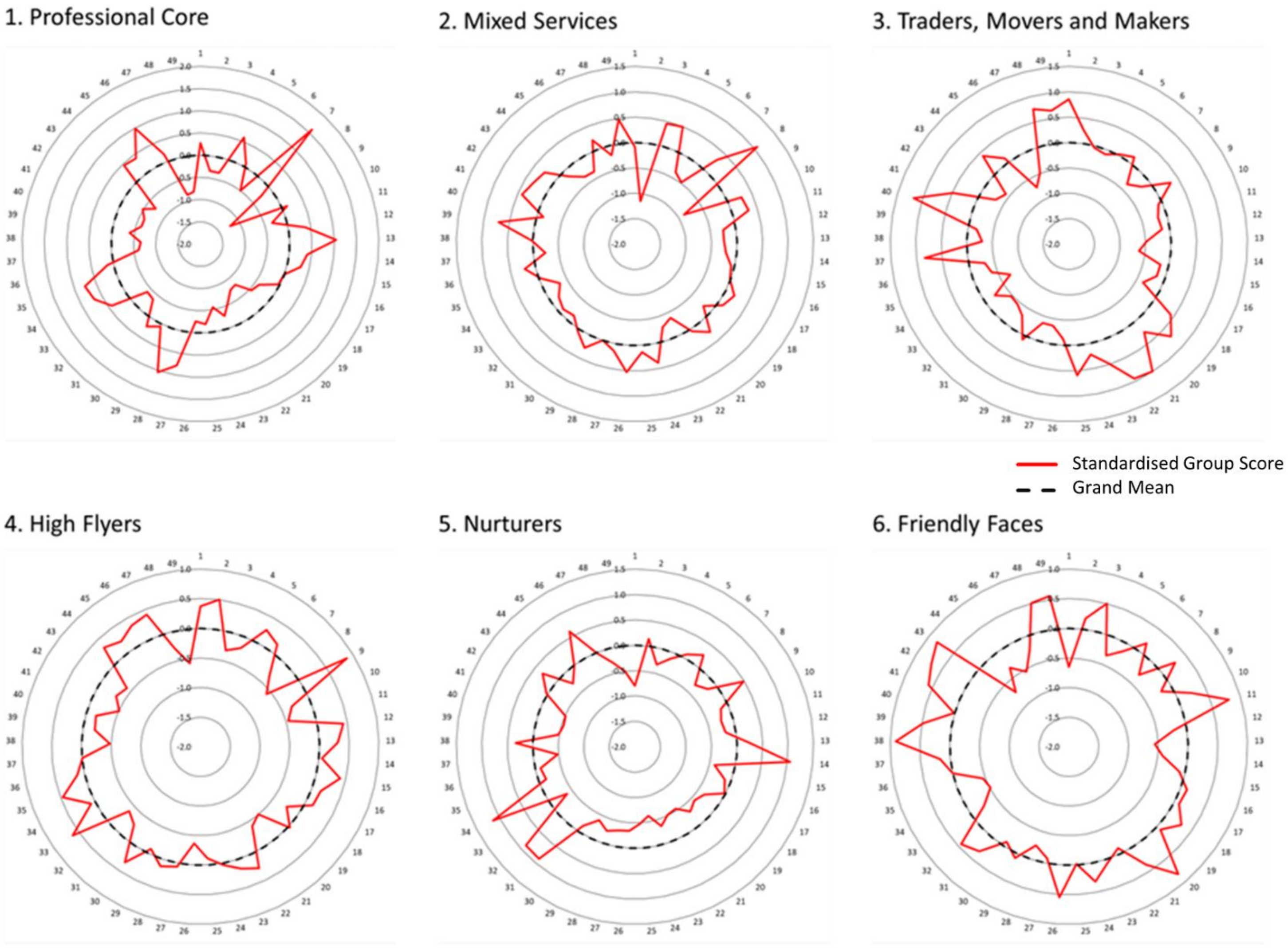

Notes

Standardised z-scores were plotted relative to the grand mean score of all NUTS3 units in the analysis. The unbroken line represents the mean trend across different indicators, while the hashed line at zero denotes the grand mean score of each of the groups. When the unbroken line surpasses the grand mean score, the metric performs above the overall average for that cluster. Conversely, if the unbroken line falls below the grand mean line, the metric performs below the overall average for that cluster (see Gale et al., 2016; Hincks et al., 2018).

A3: Geodemographic Labour Market Areas

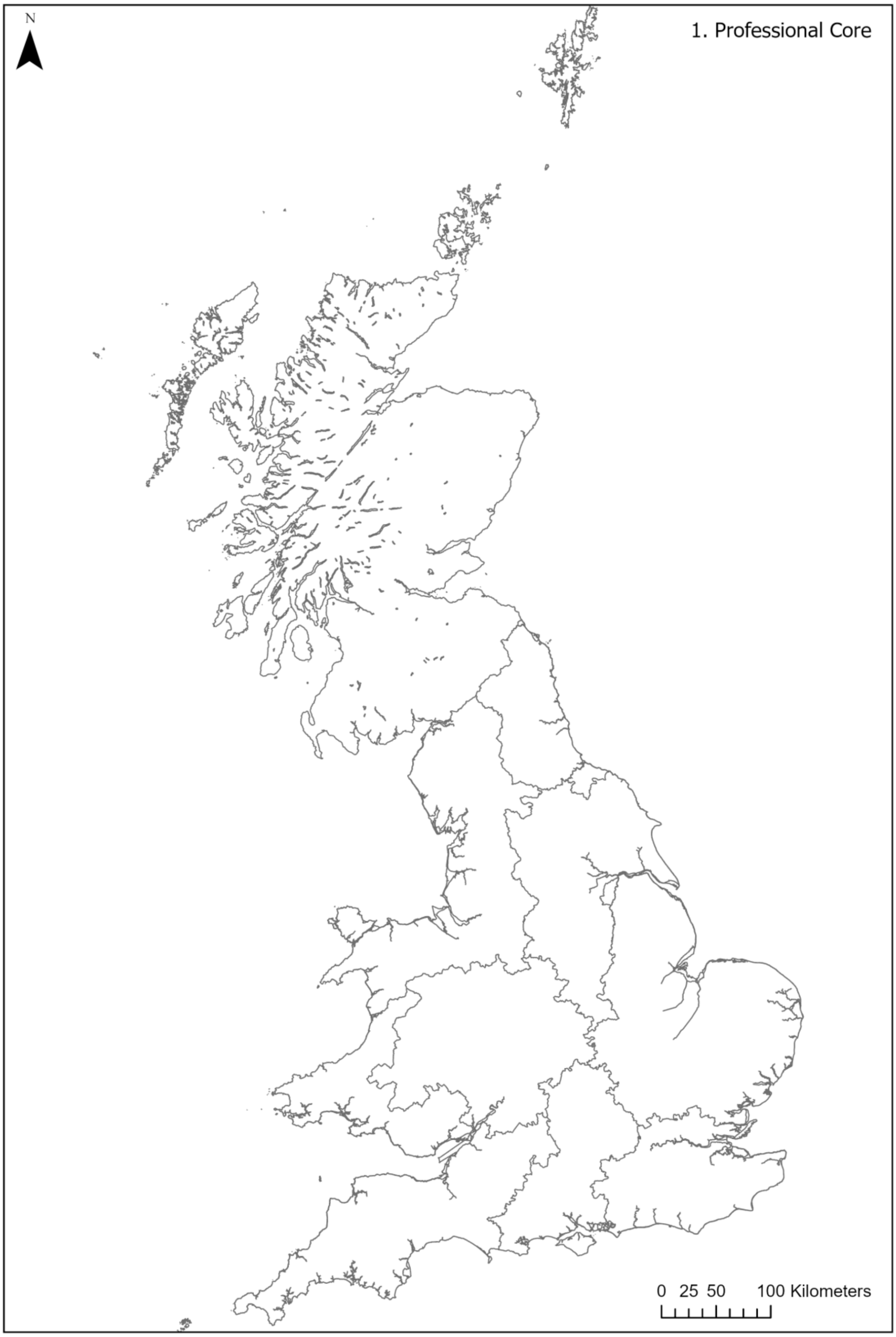

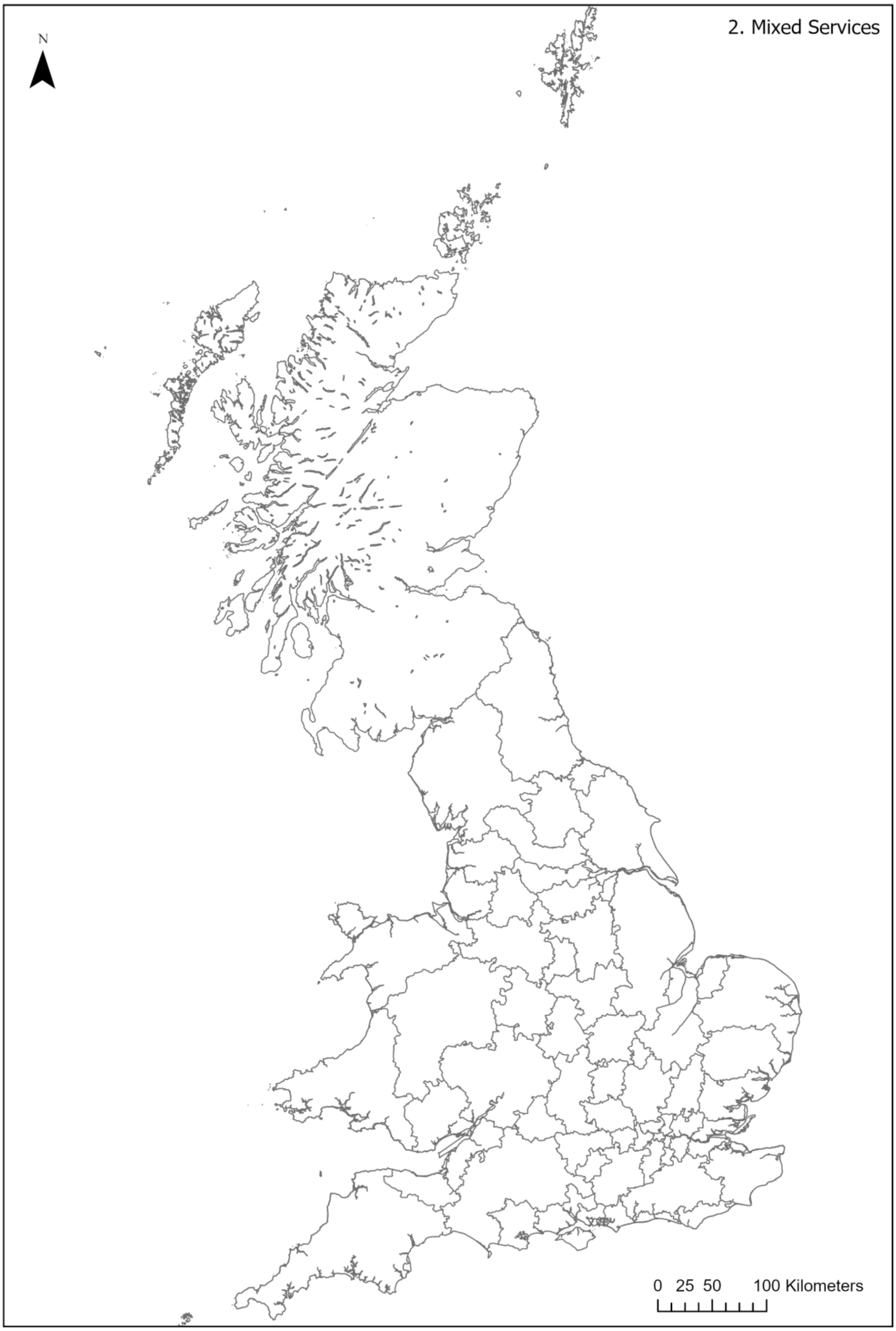
2. Mixed Services
N
0 25 50 100 Kilometers

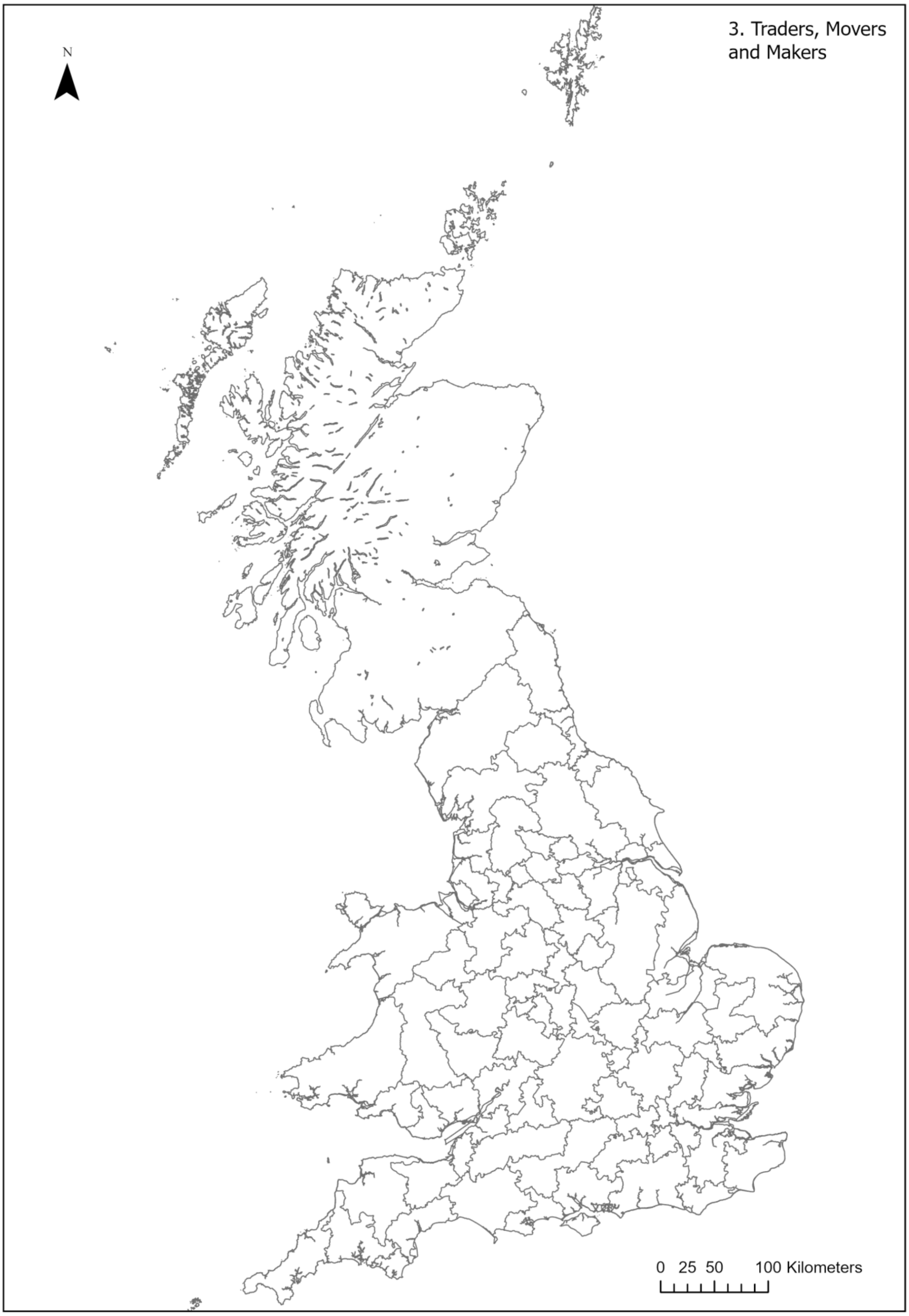
3. Traders, Movers and Makers
N
0 25 50 100 Kilometers

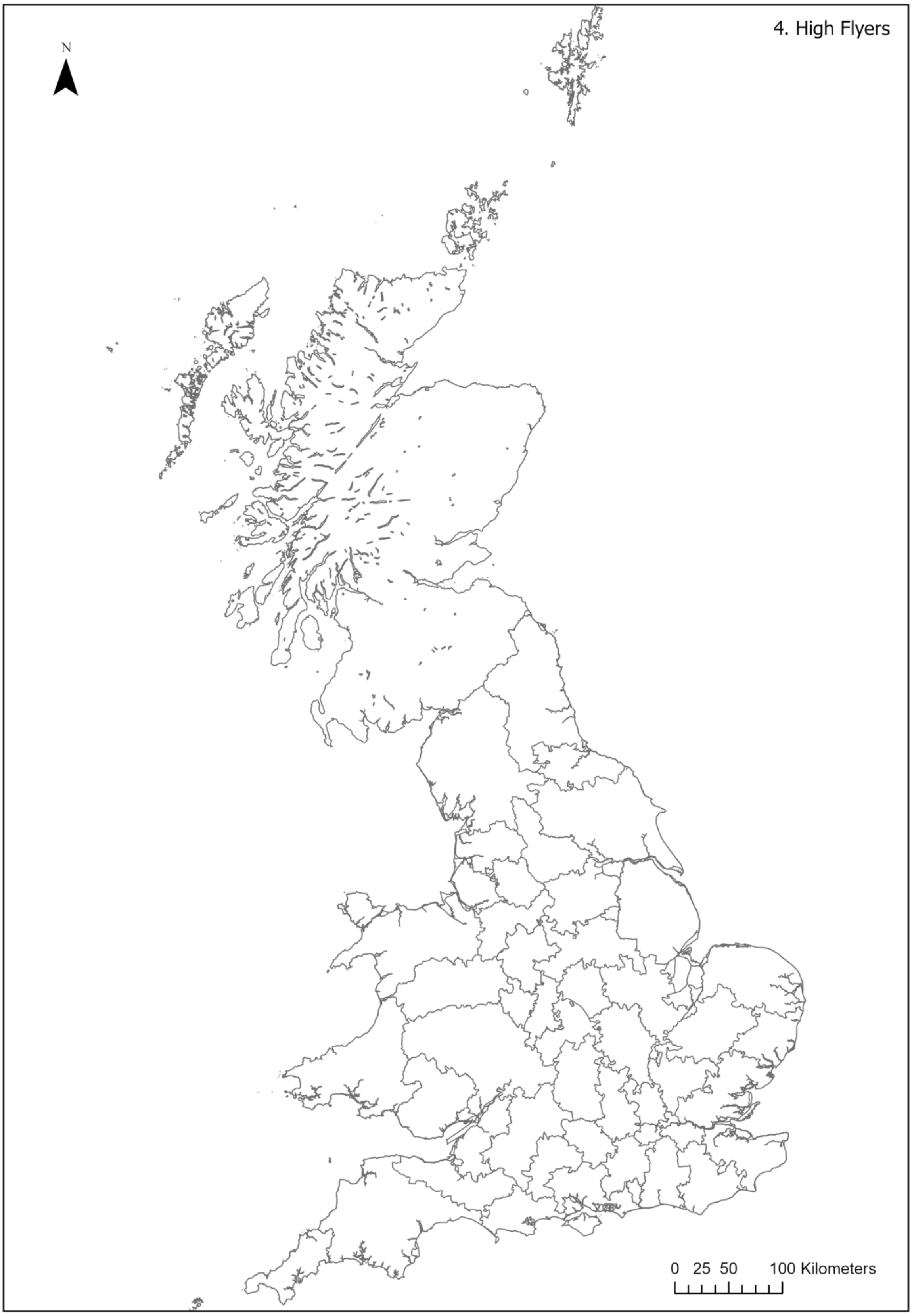
4. High Flyers
N
0 25 50 100 Kilometers

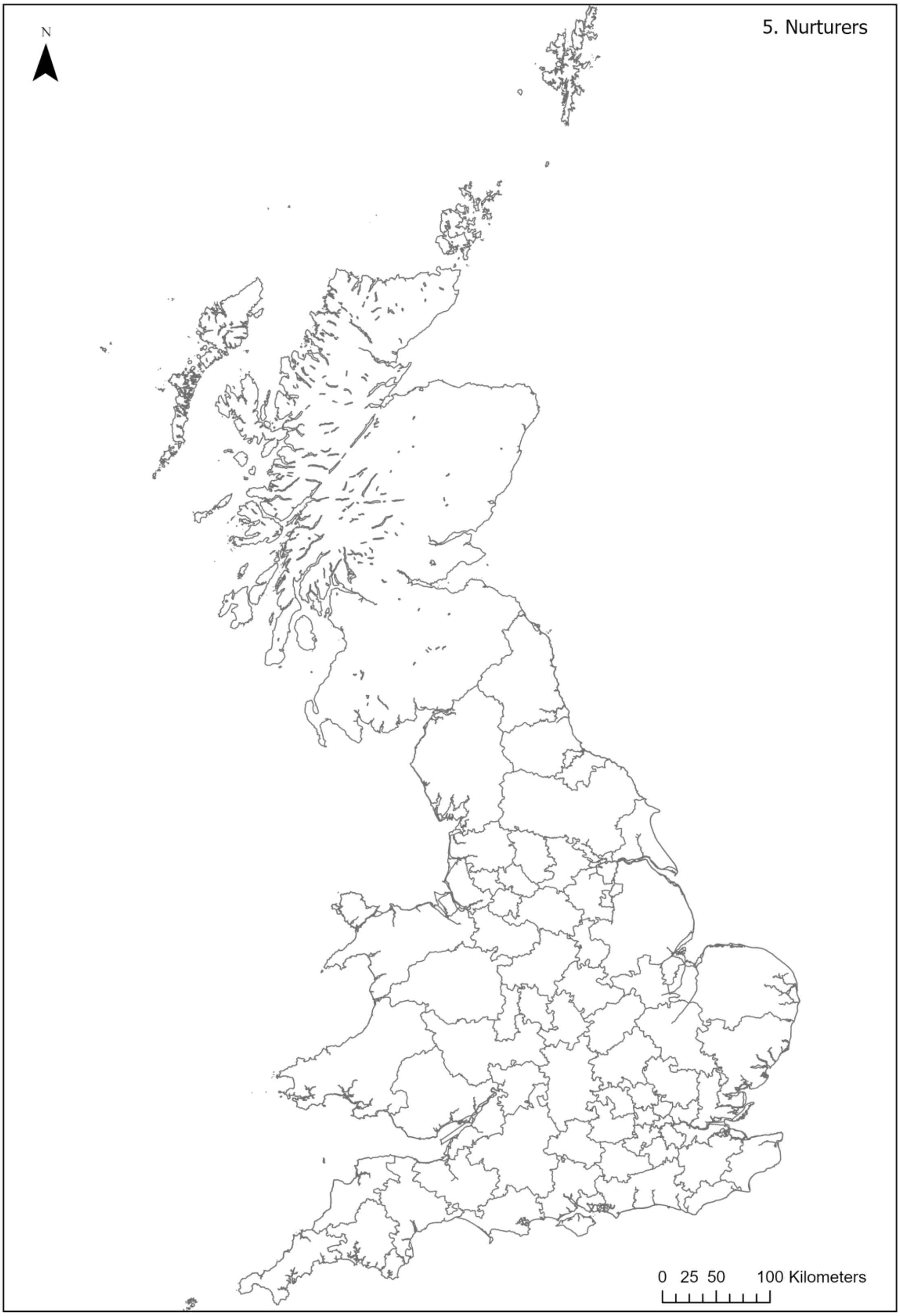
5. Nurturers
N
0 25 50 100 Kilometers

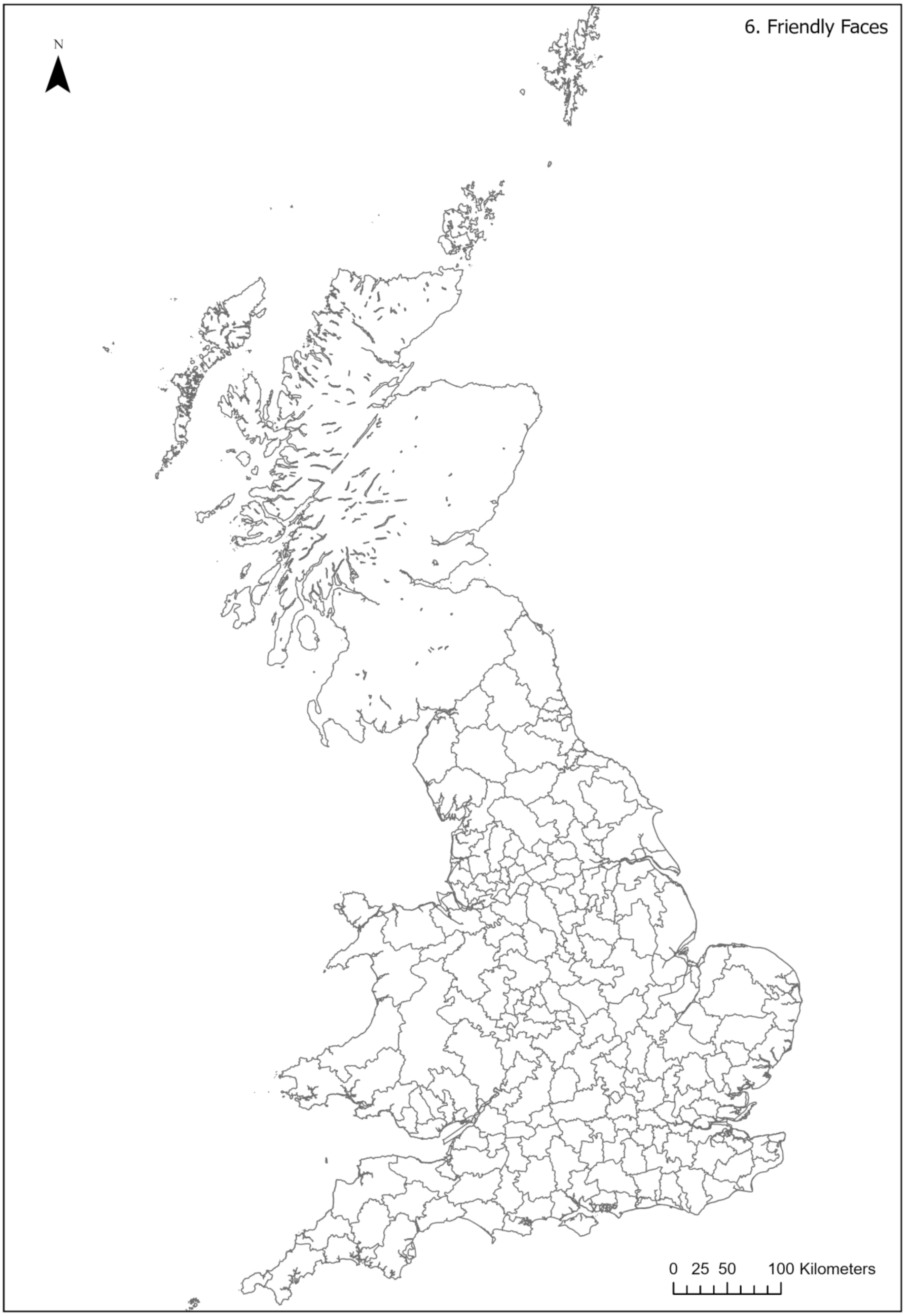
6. Friendly Faces
N
0 25 50 100 Kilometers

A4: Densification and mobility across geodemographic LMAs

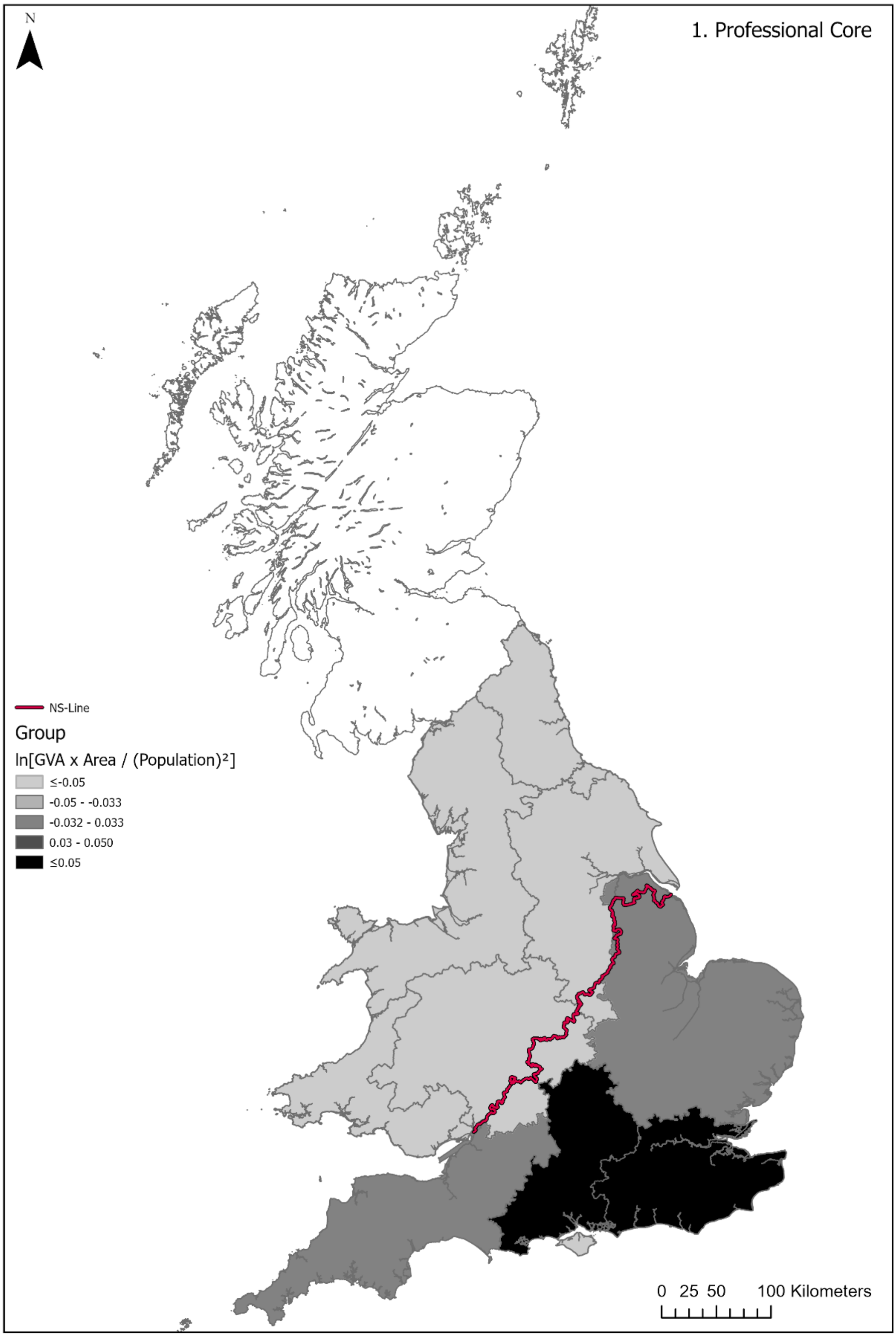

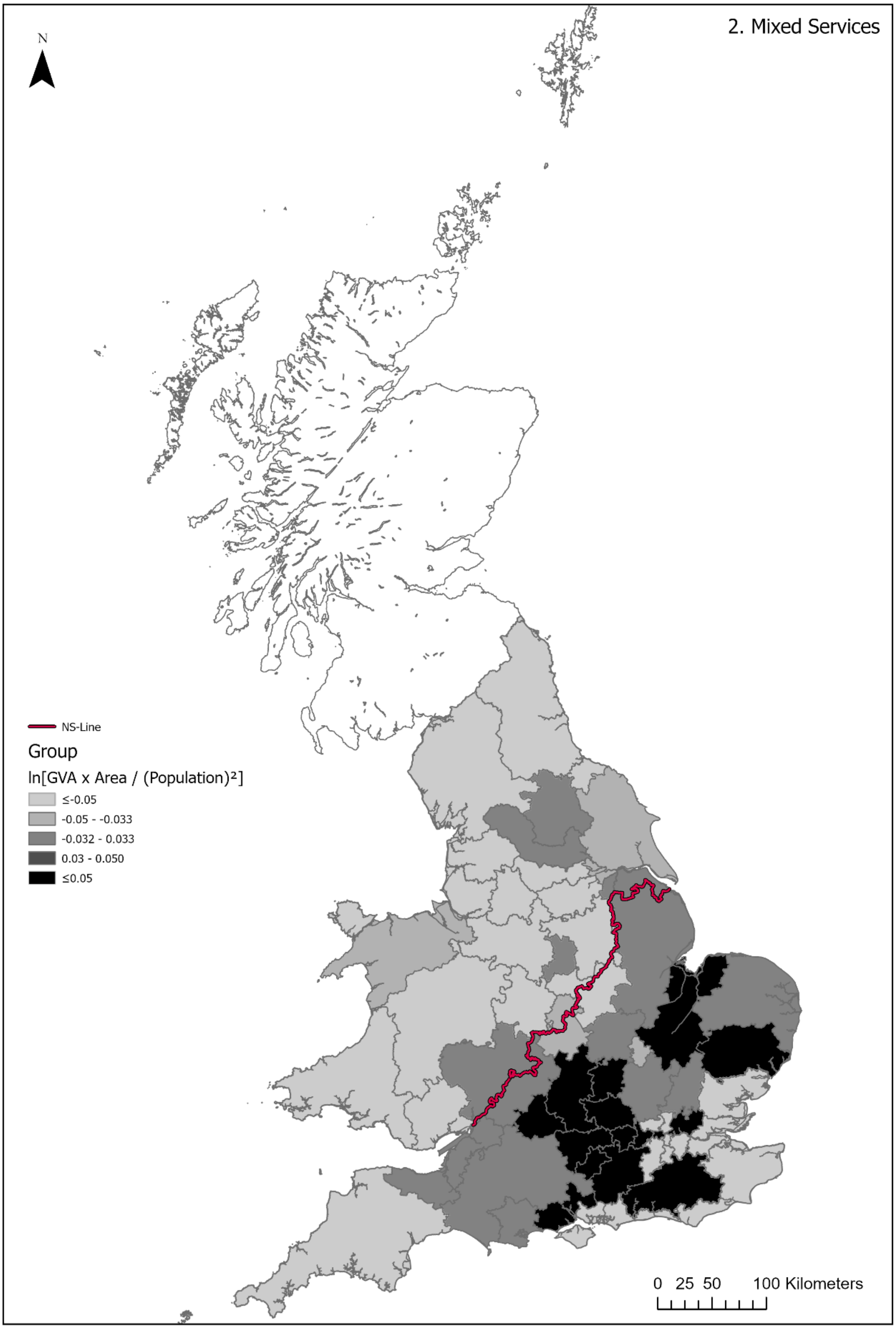
2. Mixed Services
N
NS-Line
Group
ln[GVA x Area / (Population)²]
≤-0.05
-0.05 - -0.033
-0.032 - 0.033
0.03 - 0.050
≤0.05
0 25 50 100 Kilometers

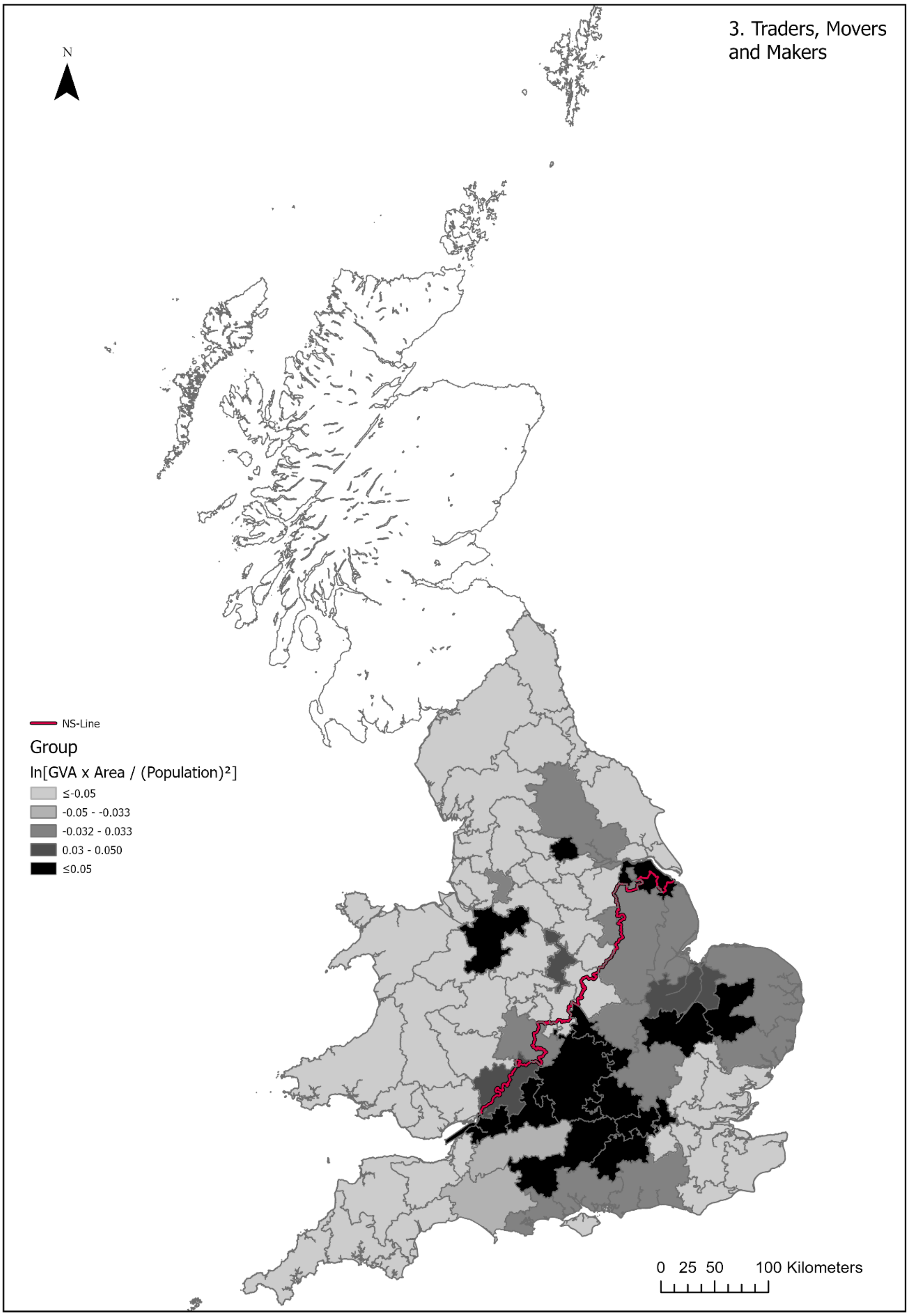
3. Traders, Movers and Makers
N
NS-Line
Group
ln[GVA x Area / (Population)²]
≤-0.05
-0.05 - -0.033
-0.032 - 0.033
0.03 - 0.050
≤0.05
0 25 50 100 Kilometers

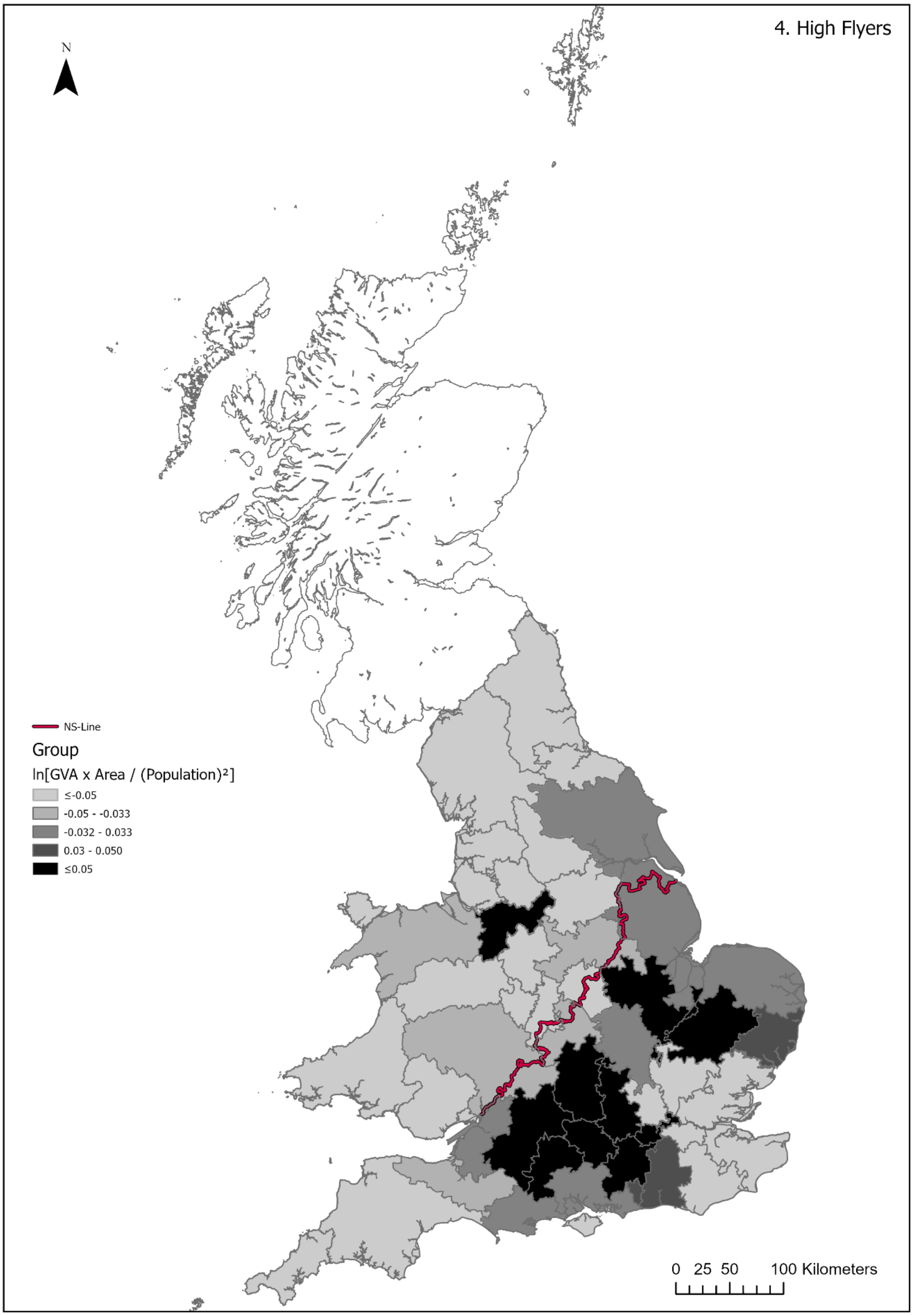
4. High Flyers
N
NS-Line
Group
ln[GVA x Area / (Population)²]
≤-0.05
-0.05 - -0.033
-0.032 - 0.033
0.03 - 0.050
≤0.05
0 25 50 100 Kilometers

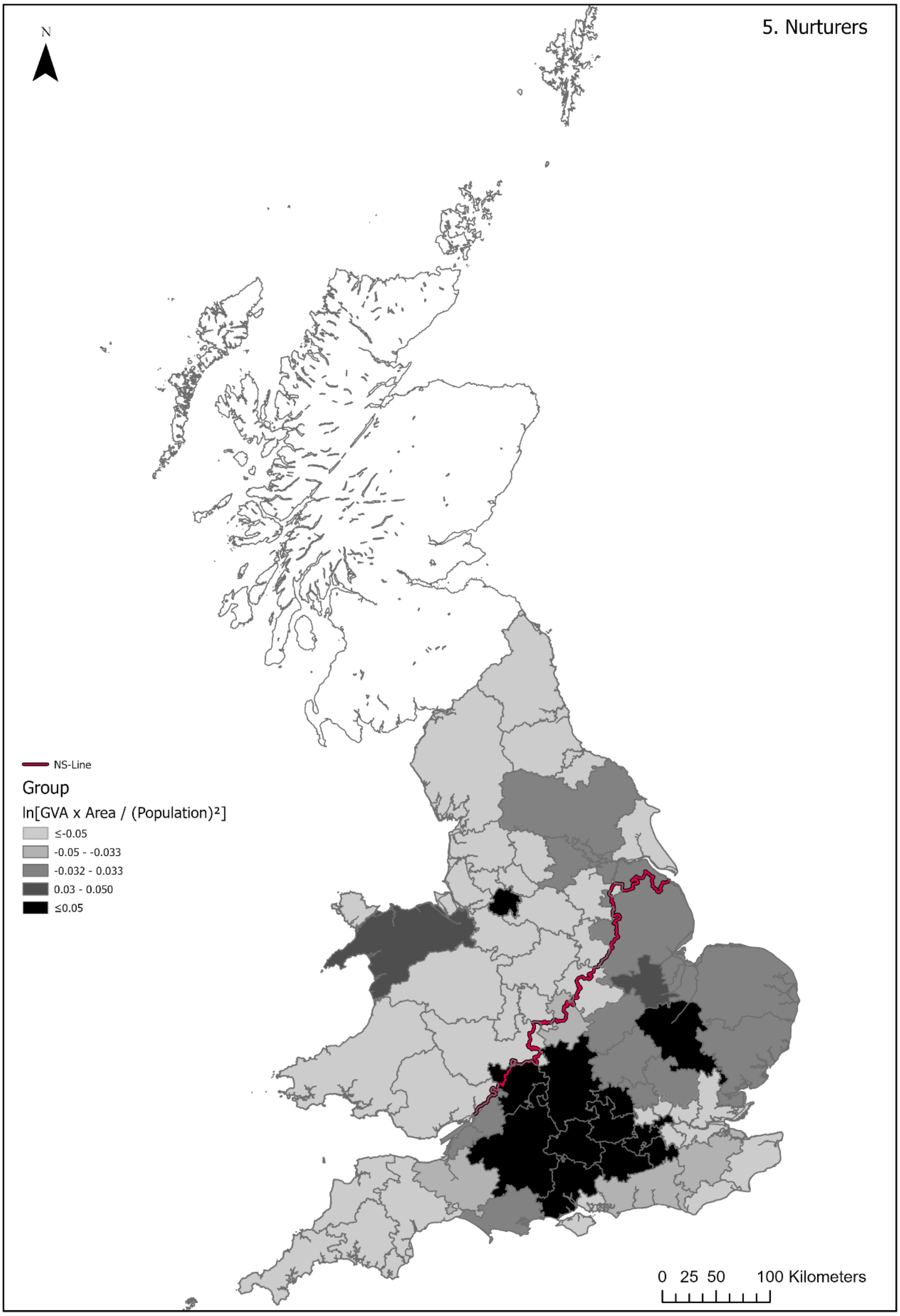
5. Nurturers
N
NS-Line
Group
ln[GVA x Area / (Population)²]
≤-0.05
-0.05 - -0.033
-0.032 - 0.033
0.03 - 0.050
≤0.05
0 25 50 100 Kilometers

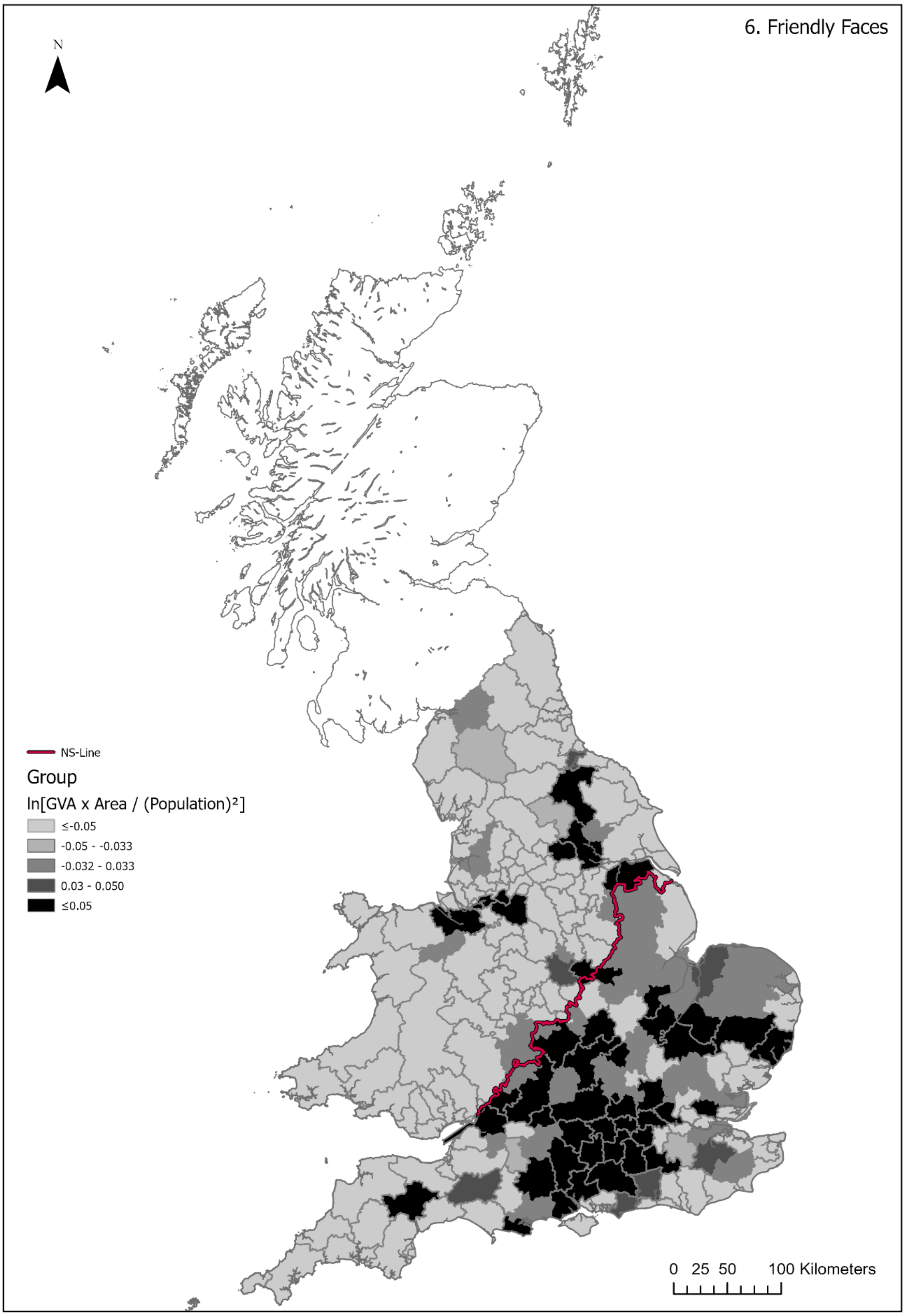
6. Friendly Faces
N
NS-Line
Group
ln[GVA x Area / (Population)²]
≤-0.05
-0.05 - -0.033
-0.032 - 0.033
0.03 - 0.050
≤0.05
0 25 50 100 Kilometers

References

Gale, C.G., Singleton, A.D., Bates, A.G. and Longley, P.A. (2016) Creating the 2011 area classification for output areas (2011 OAC). Journal of Spatial Information Science, 12, 1–27.

Hincks, S., Kingston, R., Webb, B. and Wong, C. (2018) A new geodemographic classification of commuting flows for England and Wales. International Journal of Geographical Information Science, 32(4), 663-684.
